# metGWAS 1.0: an R workflow for network-driven over-representation analysis between independent metabolomic and meta-genome-wide association studies

**DOI:** 10.1093/bioinformatics/btad523

**Published:** 2023-08-23

**Authors:** Saifur R Khan, Andreea Obersterescu, Erica P Gunderson, Babak Razani, Michael B Wheeler, Brian J Cox

**Affiliations:** Department of Medicine (Cardiology), University of Pittsburgh, Pittsburgh, PA 15261, United States; University of Pittsburgh Medical Center, Pittsburgh, PA 15213, United States; Pittsburgh VA Medical Center, Pittsburgh, PA 15240, United States; Department of Physiology, University of Toronto, Toronto, ON M5S 1A8, Canada; Toronto General Research Institute (Advanced Diagnostics), Toronto, ON M5G 2C4, Canada; Department of Physiology, University of Toronto, Toronto, ON M5S 1A8, Canada; Division of Research, Kaiser Permanente Northern California, Oakland, CA 94612, United States; Kaiser Permanente Bernard J. Tyson School of Medicine, Pasadena, CA 91101, United States; Department of Medicine (Cardiology), University of Pittsburgh, Pittsburgh, PA 15261, United States; University of Pittsburgh Medical Center, Pittsburgh, PA 15213, United States; Pittsburgh VA Medical Center, Pittsburgh, PA 15240, United States; Department of Physiology, University of Toronto, Toronto, ON M5S 1A8, Canada; Toronto General Research Institute (Advanced Diagnostics), Toronto, ON M5G 2C4, Canada; Department of Physiology, University of Toronto, Toronto, ON M5S 1A8, Canada; Department of Obstetrics and Gynaecology, University of Toronto, ON M5G 1E2, Canada

## Abstract

**Motivation:**

The method of genome-wide association studies (GWAS) and metabolomics combined provide an quantitative approach to pinpoint metabolic pathways and genes linked to specific diseases; however, such analyses require both genomics and metabolomics datasets from the same individuals/samples. In most cases, this approach is not feasible due to high costs, lack of technical infrastructure, unavailability of samples, and other factors. Therefore, an unmet need exists for a bioinformatics tool that can identify gene loci-associated polymorphic variants for metabolite alterations seen in disease states using standalone metabolomics.

**Results:**

Here, we developed a bioinformatics tool, metGWAS 1.0, that integrates independent GWAS data from the GWAS database and standalone metabolomics data using a network-based systems biology approach to identify novel disease/trait-specific metabolite-gene associations. The tool was evaluated using standalone metabolomics datasets extracted from two metabolomics-GWAS case studies. It discovered both the observed and novel gene loci with known single nucleotide polymorphisms when compared to the original studies.

**Availability and implementation:**

The developed metGWAS 1.0 framework is implemented in an R pipeline and available at: https://github.com/saifurbd28/metGWAS-1.0.

## 1 Introduction

Biochemical reactions catalysed by enzymes give rise to a diverse array of small molecules known as metabolites (Suhre *et al.* 2017, Ni *et al.* 2020). These metabolites represent the intricate integration of biological states influenced by environmental and lifestyle factors (Rattray *et al.* 2018). These metabolites interact with diverse proteins and serve critical roles as nutrients, building blocks, receptor ligands, transcriptional cofactors, genetic activators, and suppressors that modulate biological systems' adaptive responses (Rinschen *et al.* 2019). In many diseases, there is a disruption of metabolic homeostasis. In particular, metabolic changes are frequently observed before the onset of type 2 diabetes and cardiovascular disease (Visscher *et al.* 2012, Gallagher and Chen-Plotkin 2018, Rattray *et al.* 2018, Khan *et al.* 2019, 2020, Liu and Montgomery 2020, Sirdah and Reading 2020). Recent advances in high throughput methods to profile bodily fluids and tissue metabolomes have provided a wealth of information regarding metabolic health. Robust methods, including mass spectrometry and nuclear magnetic resonance, that quantify the metabolome are expanding and becoming universal tools to identify disease pathology (Tzoulaki *et al.* 2014). This is greatly facilitated by the ample availability of blood plasma, saliva, and urine, which are easily obtained and contain a rich diversity of metabolites (Wishart 2019).

Genome-wide association studies (GWAS) are critical for discovering genetic predispositions (i.e. disease-risk loci and risk-alleles) for most diseases (Dehghan 2018). However, many disease variants have small effects and are difficult to detect, even with large cohorts of individuals. On the other hand, variant associations with metabolite levels are stronger (Gieger *et al.* 2008). The integrated method, metabolomics-GWAS, aims to discover the genetic predispositions for metabolic alterations that may lead to disease (Rhee *et al.* 2013, Al-Khelaifi *et al.* 2019). While a successful strategy, the combined technological complexity and high cost are barriers to expanded use of metabolomics-GWAS. Notably, many standalone metabolomics and GWAS studies investigating the same diseases exist in independent cohorts. An *in silico* platform that allows the merging of independent GWAS and metabolomics data to identify statistical relationships could facilitate the discovery of new target candidates for diagnostic and therapeutic interventions.

Motivated to fill this need, we created metGWAS 1.0, a standalone R pipeline that integrates independent GWAS and metabolomics datasets using a network-based systems biology approach. Enriched metabolites are mapped to catalysing and interacting protein-coding genes through the Human Metabolome Database (https://hmdb.ca/) and the UniProt database (www.uniprot.org). A network representation of the GWAS Catalog, which consolidates hundreds of published GWAS studies, is used to identify metabolite-interacting genes potentially related to the phenotype of interest. Lastly, a hypergeometric test is used to test the observed intersection of the metabolite and genomic datasets.

## 2 Materials and methods

**Figure 1.**
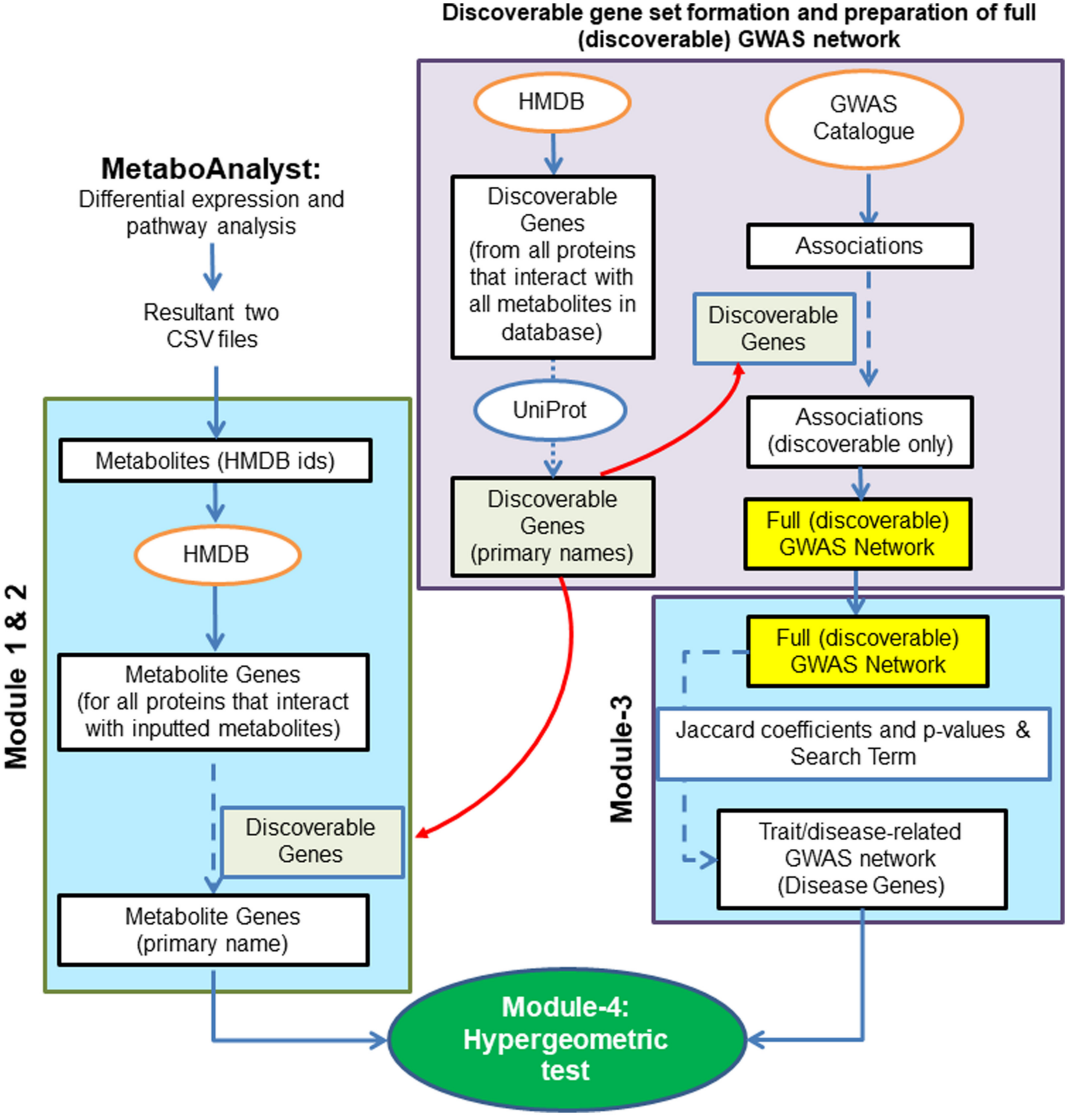
Workflow diagram for metGWAS 1.0 analysis. MetaboAnalyst is used to calculate enriched metabolites and KEGG pathways. Modules 1 and 2 convert metabolites from enriched KEGG pathways into interacting proteins and genes. Module 3 filters the network model of the GWAS Catalog for a user-supplied key-term and significance threshold. Module 4 performs a hypergeometric test by comparing the enriched metabolite interacting genes with the trait/disease-related GWAS network. Saved objects associated with the workflow are highlighted in yellow, while the steps taken to create them are shown in purple boxes. The steps that are executed each time the workflow runs are indicated by blue boxes.

The workflow presented here integrates MetaboAnalyst tools into custom functions within *R* provided as metGWAS 1.0 (Fig. 1). The user does not need any prior knowledge of *R*. We include the R-script for running the workflow and a user manual with a tutorial and technical information (Supplementary Material S1). To facilitate mapping metabolites to genes, we limit data to only those genes coding for proteins that interact with a metabolite, as annotated through the Human Metabolome Database (https://hmdb.ca/). This workflow has been described below step-by-step.

### 2.1 MetaboAnalyst: differential expression and pathway analysis

The first step of the workflow starts with differential expression analysis of an appropriately preprocessed (i.e. missing value estimation, normalization, and data transformation) standalone metabolomic dataset using the MetaboAnalyst platform (https://www.metaboanalyst.ca/), a well-known bioinformatics tool for metabolomics analysis. The differentially expressed metabolites are mapped to human metabolite database (HMDB) identifiers and linked to the *Homo sapiens* KEGG pathways library of MetaboAnalyst. Next, hypergeometric and relative-betweenness centrality tests identify any over-represented KEGG pathways. MetaboAnalyst will generate two output CSV files, named ‘name_map’ and ‘pathway_results’, which will be required when initiating Module 1 of this metGWAS 1.0 platform. We recommend separating downregulated and upregulated metabolites’ HMBD IDs to identify downregulated and upregulated KEGG pathways.

### 2.2 Module 1: identification and annotation of the corresponding metabolites for the selected over-represented KEGG pathways

In Module 1, using the CSV files ‘name_map’ and ‘pathway_results’, the user specifies an FDR *P*-value threshold and impact (the relative-betweenness centrality, valued from 0 to 1) to filter the list of MetaboAnalyst identified KEGG pathways. We recommend using FDR ≤ 0.05 to select the significantly altered KEGG pathways. Once the KEGG pathway(s) of interest is selected, Module 1 annotates metabolites belonging to the chosen KEGG pathway through a call-out to the KEGG database. Metabolites are annotated as HMDB ids. Large numbers of pathways (12 s) and metabolites (100 s) can take an extended processing time (20–30 min).

### 2.3 Module 2: identification of the metabolite-interacting proteins and their gene symbols

Module 2 maps metabolites to interacting proteins by searching metabolites’ HMDB ids in the HMDB database (https://hmdb.ca/). The list of interacting proteins is annotated as UniProt IDs (https://www.uniprot.org/) and mapped to current gene symbols. Only human genes and associated gene symbols are used, as the GWAS Catalog is based on human studies.

### 2.4 Module 3: trait/disease-related GWAS network preparation from the GWAS Catalog

The entire GWAS Catalog, a GWAS database, is filtered to only include genes with annotated metabolite interactions through the Human Metabolome Database (https://hmdb.ca/). We provide this filtered GWAS Catalog network as a prebuilt network (Supplementary Material S1). We provide code to rebuild the network using updated GWAS Catalogs, which takes 3–4 h on a standard computer. If the user chooses to update the GWAS network, they can download an updated version of the GWAS Catalog, which the workflow will use to create a network.

To construct the GWAS network (as of December 2019), each study/trait from the table is used as a node in the network. The nodes are annotated with PubMed ID, study title, study trait (phenotype), and the reported significant genes. Next, the genes annotated to the nodes are filtered to contain only those found in the HMDB (referred to as the discoverable gene set) (Fig. 1, detail is given in the technical document in Supplementary Material S1). Filtering to include only metabolite interacting genes ensures that both gene sets (metabolic and GWAS) are derived from the same background set of genes. To build edges between nodes, the Jaccard coefficient (a scoring system that measures similarity between two sets of objects) and associated *P*-values are calculated between all pairwise nodes. Significant edges (based on user-supplied thresholds for Jaccard coefficients and *P*-values) are kept.

**Figure 2.**
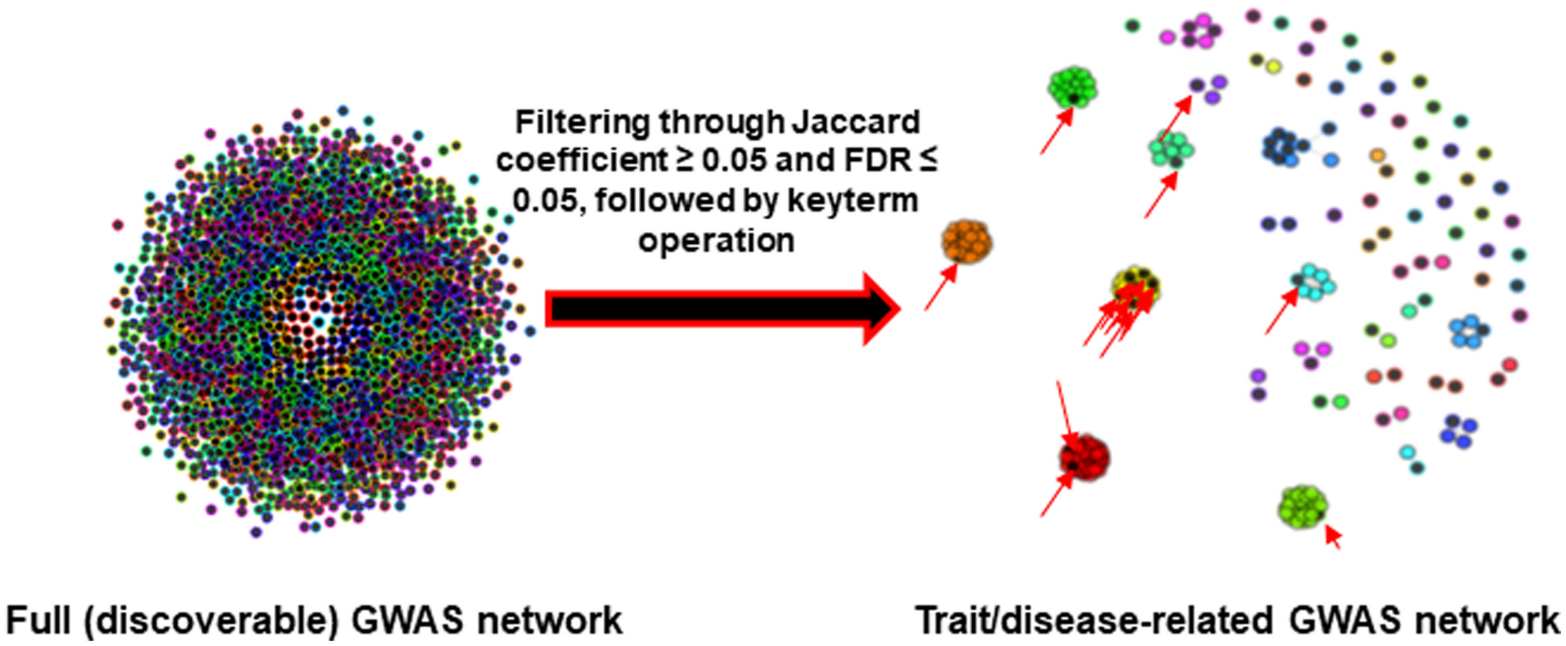
Representation of a trait/disease-related GWAS network generated by Module 3 from the full (discoverable) GWAS network. Each node represents a study/trait, and nodes are connected when there is an overlap between their associated genes. On the left, the full network, without any filtering is shown. After filtering, on the right, primary nodes identified through the key term search are depicted in black (highlighted with arrows). Nearest neighbours are represented by coloured nodes. This specific trait/disease-related GWAS network was constructed using case study 2.

**Figure 3.**
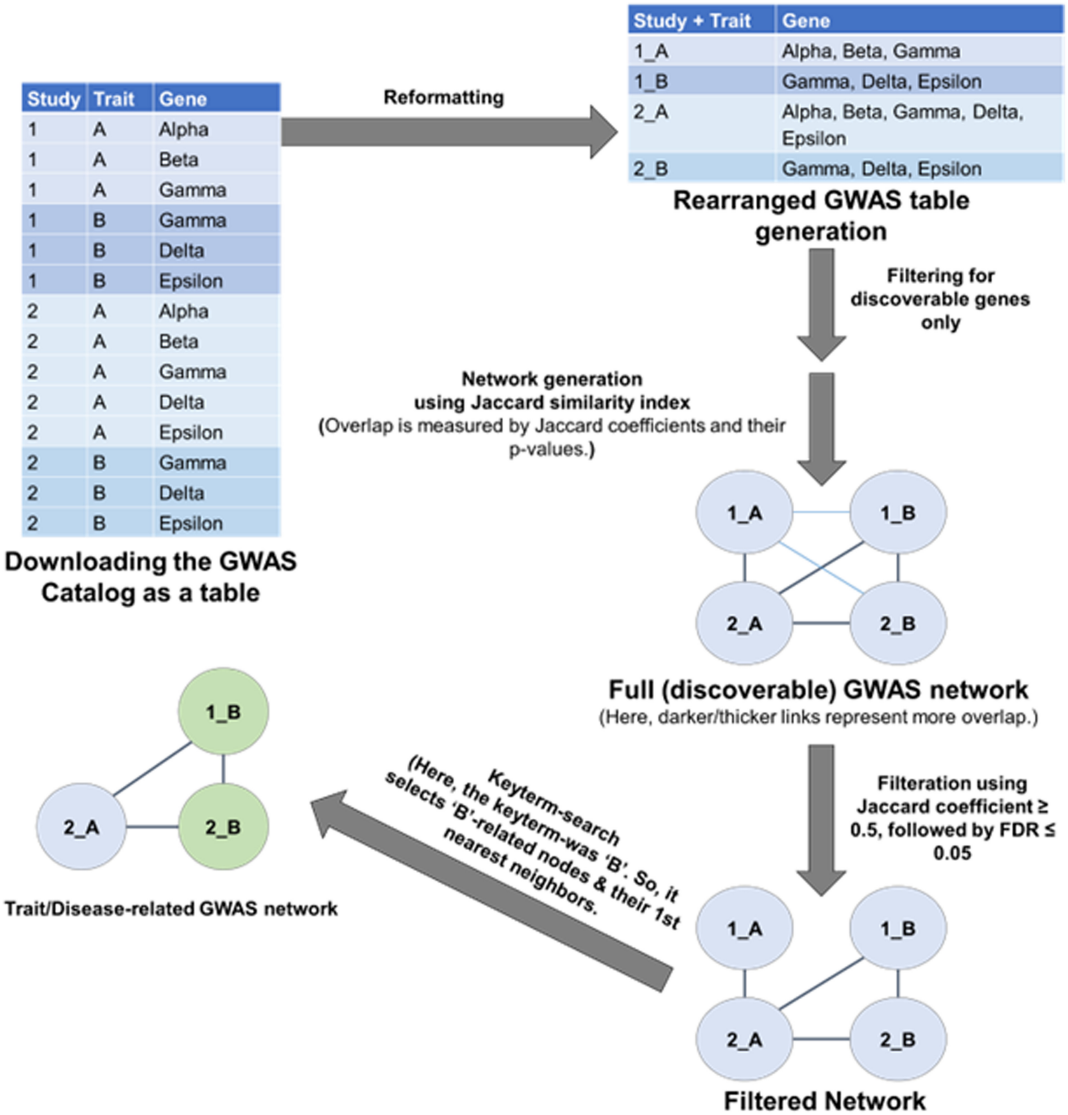
Schema of trait/disease-related GWAS-network creation. The GWAS Catalog is downloaded as a table (represented by the table on the top left). The table includes information on PubMed ids, study titles, reported traits, SNPs, and mapped genes. We work with the mapped genes, not directly with the SNPs. The table is then collapsed into a smaller table (top right). Here, each study+trait combination seen in the top-left table represents a unique row in the new table where all the gene associations have been listed together. Each row in this table will be a node in the network. These genes are first filtered using a discoverable gene set. Next, a network was built by calculating a matrix of Jaccard coefficients measuring the overlapping genes between these discoverable (or filtered) nodes. If there is a significant (according to user-supplied thresholds) overlap between the genes represented by the nodes, then there is a link between the nodes. Next, the workflow selects nodes with a key-term in their study title or trait name and the nodes' first nearest neighbours under the assumption that neighbours with significant overlap are also of interest. All other nodes are removed from the network, and the list of genes of the filtered network model serves as the trait-related gene set.

A keyword search of the GWAS Catalog identifies primary nodes, which are annotated with the keyword as part of the study title or the reported trait. This process is illustrated in Figs 2 and 3. Next, the first-degree neighbour nodes are selected to ensure GWAS studies with related phenotypes are captured. The workflow allows the user to set thresholds for Jaccard coefficients and FDR-adjusted *P*-values for nearest neighbour selection. We considered overlap significant if the Jaccard coefficient ≥ 0.5 (a Jaccard coefficient of 0.5 is considered very conservative) and the FDR-corrected *P*-value ≤ .05. A lower Jaccard coefficient value would increase the chance of network inflation with unrelated genes and studies, increasing the false discovery. The details of the trait/disease related GWAS network (or disease gene set/network) construction are illustrated in Fig. 3.

### 2.5 Module 4: over-representation analysis for genetic predisposition

Module 4 calculates the *P*-value of an overrepresentation test on the intersection of the metabolite interacting genes from Module 2 and genes of the trait/disease-related GWAS network from Module 3. Over-representation is calculated using the hypergeometric test. The total population is all genes in the discoverable gene set. The number of successes (disease) is defined as genes from the trait/disease related GWAS network. The draw size is the number of metabolite-interacting genes; observed successes are the GWAS genes within the draw. As we are conducting a single test, no correction method is needed. Significant over-representation suggests a putative causal relationship with the polymorphism containing gene loci and metabolite change, potentially predisposing to disease development.

## 3 Results

### 3.1 metGWAS 1.0 platform case assessments

The metGWAS 1.0 platform was applied to two case separate studies to evaluate performance as proof of concept. Case studies 1 and 2 were obtained from published metabolomics-GWAS studies including paired metabolomics and GWAS data. We treated the metabolomics data of these metabolomics-GWAS studies as standalone datasets and linked the metabolites to interacting genes from the HMDB. We also created trait/disease-related GWAS networks for the phenotypes of interest (using Jaccard coefficient ≥ 0.5 and FDR-corrected *P*-value ≤ .05 as threshold). We compared our findings to those of the studies to determine if previously identified gene loci could be rediscovered, as well as novel metabolite-to-phenotype gene interactions. Novel gene associations between metabolites and disease are possible, because the GWAS Catalog study populations are independent of the case study populations, and the algorithm can be used to assess a similar phenotype or trait (e.g. heart disease).

#### 3.1.1 Case study 1: the description of the original metabolomics-GWAS dataset

A metabolomics dataset (provided in Supplementary Material S2) for case study 1 was extracted from Supplementary Table S1 of a metabolomics-GWAS of human plasma, designed to identify the link between the lipidome and cardiovascular disease (CVD) (Tabassum *et al.* 2019). A total of 42 unique metabolites with significant (*P*-value of < 1.5 × 10^−9^) genetic associations were found (Tabassum *et al.* 2019). We excluded three of the 42 metabolites as they were not mappable to an HMDB id (i.e. nonspecific metabolites belonging to total ceramides, total sphingomyelins, and total phosphatidylethanolamines). For the 39 remaining metabolites, the study reported associations with 11 genes, five of which were also associated with CVD.

#### 3.1.2 Case study 1: the results of metGWAS 1.0 execution

We applied metGWAS to the 39 metabolites and identified the KEGG pathway glycerophospholipid metabolism as significantly enriched via MetaboAnalyst (Table 1). Next (in Module 2), metGWAS mapped 86 interacting protein-coding genes to the study metabolites present in the glycerophospholipid metabolism pathway. This gene set serves as metabolite-interacting genes. Next (in Module 3), metGWAS filtering of the full GWAS network with the key term ‘Cardiovascular disease’ identified 84 nodes containing 189 genes. The 189 genes annotated to the cardiovascular disease-related GWAS subnetwork were then compared to the metabolite gene set by a hypergeometric test (in Module 4). There was a significant overlap of metabolite-interacting human genes of the ‘glycerophospholipid metabolism’ pathway with the cardiovascular disease-related GWAS subnetwork (*P *=* *.0028). Specifically, the nine overlapping genes were APOA5, PLA2G5, PLA2G2D, PLA2G2E, PLA2G2F, LRAT, PLA2G2A, PLB1, and PLA2G7. APOA5 was also one of the genes identified in the original analysis (Tabassum *et al.* 2019) associated with CVD.

**Table 1.** The results of case study 1 using the metGWAS 1.0 platform.

KEGG pathways for significant metabolites (annotated metabolites, interacting genes)	Hypergeometric test	GWAS keyword
		Cardiovascular disease
		(35 primary nodes; 49 neighbouring nodes; 189 genes)
Glycerophospholipid metabolism pathway (14 HMDB ids, 86 genes)	No. of overlapping genes	9
	Overlapping gene names	APOA5, PLA2G5, PLA2G2D, PLA2G2E, PLA2G2F, LRAT, PLA2G2A, PLB1, PLA2G7
	*P*-value	.00283

#### 3.1.3 Case study 2: the description of the original metabolomics-GWAS dataset

For case study 2, a metabolomics dataset (provided in Supplementary Material S2) was extracted from Supplementary Table S2 of a publication investigating the relationship of the urine metabolome with GWAS to identify potential detoxification variants (Suhre *et al.* 2011). Case study 2 reported 56 unique metabolites with significant genetic associations (*P* < 2.9 × 10^−5^) (Suhre *et al.* 2011) representing five genes.

#### 3.1.4 Case study 2: the results of metGWAS 1.0 execution

Using MetaboAnalyst, the 56 metabolites were enriched for the following four KEGG pathways (FDR ≤ 0.05; Table 2): ‘aminoacyl-tRNA biosynthesis’, ‘glyoxylate and dicarboxylate metabolism’, ‘glycine, serine, and threonine metabolism’, and ‘alanine, aspartate, and glutamate metabolism’. These pathways collectively represented between 5 and 10 metabolites from the study, mapping to 90–206 protein interactions. Interestingly, the gene AGXT2 was present in all four enriched pathways and was one of the five genes reported to have significant metabolite associations by the original study.

**Table 2.** The results of case study 2 using the metGWAS 1.0 platform.

KEGG pathways for significant metabolites (annotated metabolites, interacting genes)	Hypergeometric test	GWAS keyword	
		Kidney	Urine
		(59 primary nodes, 36 neighbouring nodes, 288 genes)	(8 primary nodes, 1 neighbouring node, 113 genes)
Aminoacyl tRNA biosynthesis (10 HMDB ids, 178 genes)	No. of overlapping genes	11	3
	Names	ASPG, ARG1, RPN1, GRIN2A, GSS, GATM, KYAT3, NOS3, PCCB, SLC7A6, EPRS1	AGXT2, ASRGL1, PRMT3
	*P*-value	.3457018	.723895
Glyoxylate and dicarboxylate metabolism (7 HMDB ids, 206 genes)	No. of overlapping genes	11	4
	Names	ASPG, ALDH2, CYP1A2, CYP1A1, GRIN2A, GSS, GATM, KYAT3, SLC13A3, SUCLG2, EPRS1	AGXT2, CLYBL, EGLN3, PHF8
	*P*-value	.5381508	.6284296
Glycine, serine, and threonine metabolism (6 HMDB ids, 90genes)	No. of overlapping genes	8	1
	Names	ADH1B, DMGDH, GRIN2A, GSS, GATM, KYAT3, PRRX1, SLC22A1	AGXT2
	*P*-value	.1038139	.8520755
Alanine, aspartate, and glutamate metabolism (5 HMDB ids, 129 genes)	No. of overlapping genes	5	5
	Names	ASPG, RPN1, SLC13A3, SUCLG2, EPRS1	AGXT2, CLYBL, EGLN3, PHF8, ASRGL1
	*P*-value	.8237494	.1308664

We selected the GWAS search keywords ‘Kidney’ or ‘Urine’, as they aligned with the metabolite study design, to evaluating kidney detoxification capability. This search resulted in kidney centred GWAS sub-networks of 95 nodes containing 288 genes (Table 2). The urine-centred subnetwork contained nine nodes encompassing 113 genes (Table 2). Eight genes (ADH1B, DMGDH, GRIN2A, GSS, GATM, KYAT3, PRRX1, and SLC22A1) overlapped between the metabolite-interacting human genes of the ‘glycine, serine, and threonine metabolism’ pathways and the kidney-specific GWAS network, although a hypergeometric test found this to be marginal (*P *=* *.103). Assessment of the metabolite-interacting human genes of the ‘alanine, aspartate, and glutamate metabolism’ pathway found five genes (AGXT2, CLYBL, EGLN3, PHF8, and ASRGL1) overlapping with the urine-specific GWAS network. However, the hypergeometric test was marginal (*P *=* *.13) (Table 2). Interestingly, among the overlapping genes was AGXT2, also identified in the original case study as significantly associated with variation of metabolites in urine.

### 3.2 Primary and first neighbour nodes

To understand how the first-neighbour approach facilitated the metGWAS method, we looked at how primary and first neighbour nodes contributed to the intersecting metabolic and GWAS gene sets. In case study 1, all genes were derived from first neighbour nodes, indicating that GWAS studies annotated as a cardiovascular disease were not the source of metabolic genome interactions. In contrast, the intersecting genes of case study 2 were all derived from the primary nodes. This suggests that no new information was obtained by including the first neighbour nodes.

## 4 Discussion

A metabolomics-GWAS analysis is designed to identify the interaction of polymorphic variants, particularly single nucleotide polymorphisms (SNPs), with expression levels of individual metabolites using paired samples. A series of logistic regression models are used to identify gene variants influencing metabolite levels. Many metabolomics-GWAS studies do not use a case-control cohort design but instead utilize a population of healthy individuals to observe the natural variation. It is proposed that normal variants related to changes in metabolite levels can compound with other factors (e.g. other gene variants, environmental factors, etc.) and reach a tipping point leading to disease. The advantage of an integrated metabolomics-GWAS is that it allows both direct (i.e. SNPs discovery within the exons of a gene) and indirect (i.e. SNPs discovery from nonexon sequence of a gene) associations to be observed. However, occasionally, indirect association can be found far outside of a particular gene regulatory network in metabolomics-GWAS studies. This kind of association is challenging for explanation and wet laboratory validation.

Our metGWAS 1.0 platform uses the extensive resource of independent GWAS and metabolomics studies focused on similar diseases. As these samples are unpaired, we cannot utilize the same procedure as a standard metabolomics-GWAS. Instead, we focus on annotated protein-metabolite interactions as a directly interpretable finding. Our approach arguably will identify generalizable results as both the metabolite and GWAS study population are independent.

In the first case study, metGWAS successfully reidentified one gene and discovered other potentially novel metabolites-to-gene associations. The second case study identified associations with a marginal *P*-value near .1. The case studies presented essential differences in study design. Study 1 investigated a pathology where the phenotype to gene associations are likely stronger. In study 2, all participants were considered normal, and the variation in the urine metabolome does not indicate pathology. The GWAS Catalog-derived network mainly comprises disease or phenotypes, while case study 2 did not contain any specific phenotype (i.e. healthy people). The polymorphisms found in natural variation may not be significantly associated with any pathology state. Urine is more challenging for making direct associations, as secreted metabolites may not be primarily produced by the kidney, given the liver is a major detoxification organ. In contrast, for case study 1, the assessment of CVD phenotype associations is more likely to produce overlapping results with the GWAS network that contains similar study designs. This was our observation, with a significant overlap (*P* < .05) that included a gene from the original study (APO5A).

Despite the marginal result for case study 2, some interesting findings are present. Of the five genetic loci identified (i.e. AGXT2, CLYBL, EGLN3, PHF8, and ASRGL1), belonging to the **‘**alanine, aspartate, and glutamate metabolism’ pathway, AGXT2 (i.e. reidentified gene from the original study) may be of particular interest. Within the GWAS Catalog, 16 GWAS studies identified 22 associations of AGXT2 with chronic kidney diseases using urinary metabolite measurements. Therefore, the AGXT2 gene loci may be an important candidate for further analyses of human detoxification capacity. The metGWAS platform may help narrow down the possible genetic loci for further study.

A limitation of GWAS and metabolomics-GWAS is low reproducibility between different genetic populations. For example, Suhre *et al.* (2011) (case study 2) identified five gene loci as significant in a German population (i.e. SHIP study and KORA study). Schlosser *et al.* (2020) replicated four metabolite-gene variant associations using another German cohort (i.e. GCKD study). However, Flitman *et al.* (2005) could not identify any of Shure's identified gene loci in a cohort from Lausanne, Switzerland. It is equally challenging for the metGWAS 1.0 platform since it always executes its analyses using independent GWAS and metabolite cohort populations. Moreover, the GWAS database includes different population-based studies. Therefore, the reidentification of the same gene loci of a particular metabolomics-GWAS study using the metGWAS 1.0 platform is unlikely. However, we have shown here two case studies identified gene loci from the original metabolomics-GWAS study.

Population variation is a well-known factor that undermines the reproducibility of GWAS. This may indicate why we had limited success in rediscovering the original metabolite-to-gene associations. This could conversely be advantageous, as different SNPs and genes may drive the same metabolic shifts between study populations. This is supported by case study 1, where many statistically significant novel metabolites-to-SNP associations were identified.

Metabolite-GWAS studies directly measure SNP associations with metabolite levels on paired data. However, our metGWAS platform functions at the gene level. This may challenge polymorphism-to-gene mapping of distal noncoding polymorphisms. GWAS studies often yield SNPs in noncoding regions (Liu *et al.* 2019), which are mapped to the nearest gene, but SNPs can also have long-distance regulatory effects (Võsa *et al.* 2021). Additionally, metGWAS focuses on direct protein interactions with metabolites, meaning that protein-coding and noncoding genes that function upstream or downstream from the metabolite of interest will not be identified even though they may affect metabolite levels.

Using first neighbour nodes may bring irrelevant studies as the study phenotypes or traits do not contain the keyword search terms. However, the neighbours do have a significant gene overlap, suggesting some relationships. Assessing the first neighbours may be a tool to guide a literature search and determine previously unknown genetic relationships between phenotypes. Curiously, we found that the cardiovascular disease study results were only derived from the first neighbour nodes, while the urine metabolism study results were only from primary nodes. A likely reason for these disparate findings may lie in the selected GWAS subnetwork and metabolic study designs. In case study 1, the primary cardiovascular disease nodes were connected to first neighbour nodes representing cholesterol, blood glucose, or insulin dysregulation traits. These measurements are all related to metabolic syndrome, which can increase CVD risk (Alberti *et al.* 2009). In case study 2, we observed only the primary nodes contributing to both the urine- and kidney-anchored GWAS subnetworks. This suggests the primary nodes are more related to the metabolic genetic origin, and those first neighbour nodes may relate to nonmetabolic genetic contributions.

The metGWAS 1.0 platform showed success as a discovery platform for integrating metabolite and GWAS datasets. Our results indicate that assessing studies related to specific pathologies was more successful than assessing normal variation. The network view of GWAS may lead to finding novel genetic associations between seemly unrelated phenotypes.

## Supplementary Material

btad523_Supplementary_Data

## Data Availability

All supplementary materials and the R source code to run metGWAS 1.0 as well as all necessary codes and documentation are available at https://github.com/saifurbd28/metGWAS-1.0.
