# Supplementary material for: metGWAS 1.0: an R workflow for network-driven over-representation analysis between independent metabolomic and meta-genome-wide association studies
Source: Bioinformatics. 2023 Aug 23;39(9):btad523. doi: 10.1093/bioinformatics/btad523 (PMC10491949; doi:10.1093/bioinformatics/btad523)
Supplement: btad523_Supplementary_Data [file btad523_supplementary_data.zip › Supplementary Material 1/metGWAS technical.docx]

**Technical notes on metGWAS 1.0: Network driven over-representation analysis between independent metabolomic and meta-genome wide association studies identifies gene-metabolite- relationships to phenotype.**

INTRODUCTION

The metGWAS 1.0 platform facilitates identifying possible genetic predispositions from standalone metabolite data using previously published genotyping data. The workflow is run by an R script but is interactive so that users without previous R experience can use it.

The workflow depends on data from MetaboAnalyst (1) and runs in four modules which have the following purposes:

- Module 1 – Select the over-represented KEGG pathways and annotate which differentially expressed metabolites (i.e., metabolites of interest) are in which pathways of interest.
- Module 2 – Determine which proteins interact with metabolites of interest and identify their corresponding genes. These genes are the metabolite-interacting gene set.
- Module 3 – Determine which genes are associated with a disease or trait of interest. These genes are the trait/disease gene set.
- Module 4 – Determine if metabolite genes (output from module 2) are enriched in disease genes (output from module 3).

Below, the conceptual details of modules are discussed. For instructions on how to use the workflow, please see the tutorial document in Supplementary Material 1 (“metGWAS 1.0 – Tutorial on how to map standalone-metabolomics to genome-wide association studies”).

TECHNICAL DETAILS OF MODULES

**Module 1 – *Identification and annotation of the corresponding metabolites for the selected over-represented KEGG pathways***

After conducting pathway analysis on MetaboAnalyst, users can download a file of enriched pathways and a name map that maps the input metabolites to ids in a variety of databases (such as HMDB ids from HMDB and C ids from KEGG).

Module 1 allows users to select pathways of interest from the pathway file by using FDR cut-offs and pathway impacts. It then annotates the pathways with the input metabolites that belong to them (the downloaded results from MetaboAnalyst do not contain this information). To accomplish this, module 1 searches the pathways of interest on KEGG. Pathways on KEGG have metabolites, represented by C ids, annotated to them. Module 1 uses the name map from MetaboAnalyst to identify which of the C ids were part of the inputted metabolites. It annotates these metabolites (as HMDB ids) to the original file of pathways.

**
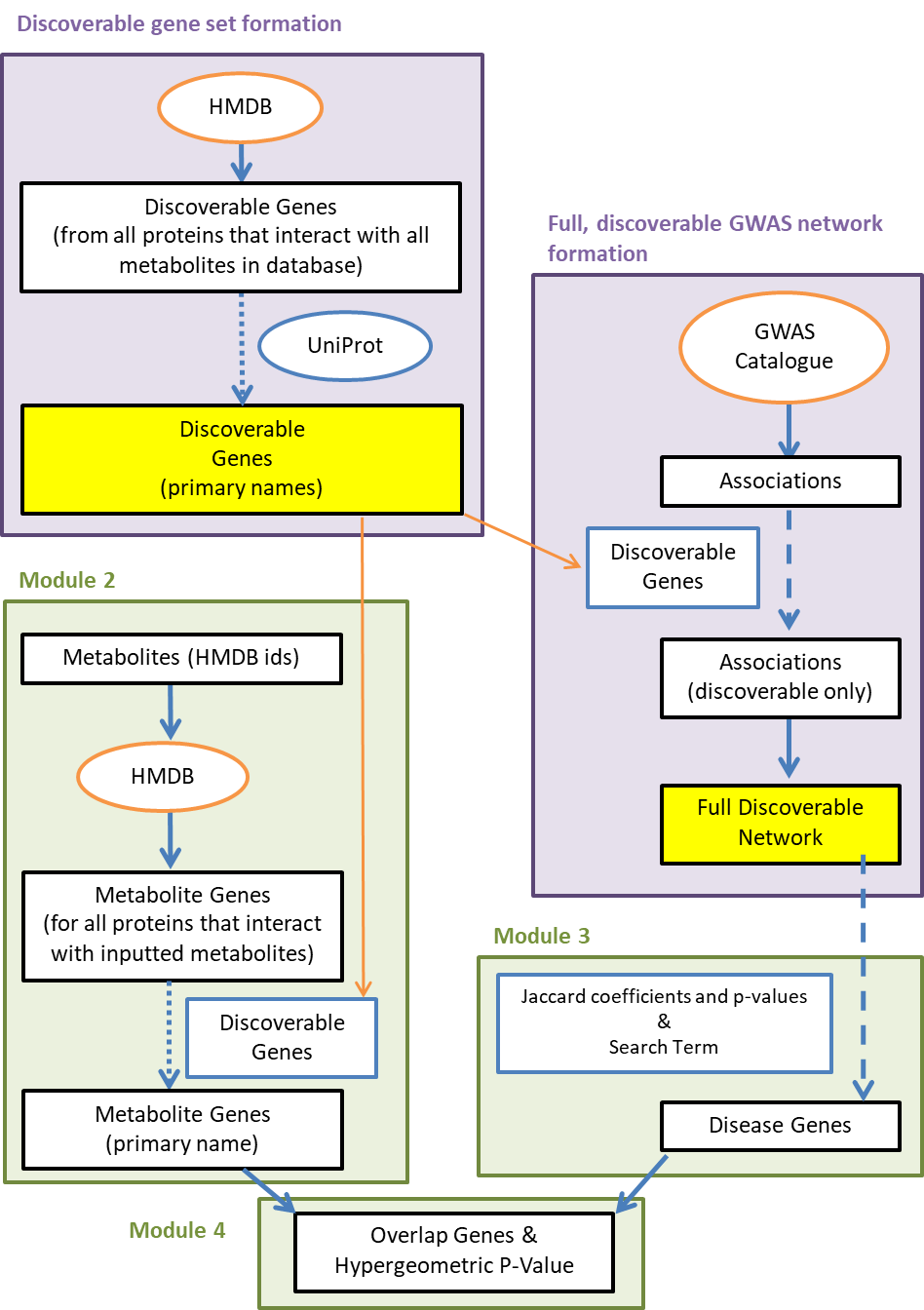
**

**Figure 1 *(previous page)*. Overview of the discoverable gene set, the full discoverable GWAS network and modules 2 to 4.** Parts of the workflow on purple backgrounds represent processes that have already been carried out. The final data from these processes (highlighted in yellow) have been saved in RData files and provided in Supplementary Material 1. For discoverable gene set formation, all genes from HMDB are considered the discoverable gene set and the gene names are standardized by UniProt. For GWAS network formation, associations from the GWAS Catalog are filted to contain only discoverable genes and then a network is created (see Fig. 2). Parts of the workflow on green backgrounds are processes that the workflow conducts every time it is run by the user. Module 2 uses HMDB to identify genes of proteins that interact with the metabolites of interest. Gene names are standardized using the discoverable gene set. Module 3 searches the GWAS network for a keyterm supplied by the user (see Fig.3) and genes from relevant nodes define the disease gene set. Module 4 compares the metabolite and disease genes with a hypergeometric test.

***Discoverable gene set formation***

The discoverable gene set is the set of genes that code for all metabolite-interacting proteins listed in HMDB (Fig. 1). It is termed the discoverable gene set because module 2 will only ever be able to detect genes present in the HMDB database. This gene set is used to limit module 3 analysis (see Module 3 section for details) to the same background gene set as module 2 so that the two modules can have their output statistically compared. Additionally, the discoverable gene set is used to standardize gene names. It is saved as an R object in the file “discoverableGenes_akaAllGenesInHMDB.RData” and is provided with the workflow in Supplementary Material 1.

The discoverable gene set was created with the UniProt and HMDB databases. The human proteome was downloaded in October 2021 from UniProt (<https://www.uniprot.org/uniprot/?query=proteome:UP000005640>). This data included protein names, UniProt ids, and gene names (both primary and synonyms). The metabolite-interacting proteome was downloaded from HMDB (all proteins XML file from <https://hmdb.ca/downloads>) in September 2021. The UniProt proteome was then filtered to contain only the proteins present in the HMDB dataset (ie metabolite-interacting proteins only). After formatting and cleaning the data, a table with a column of primary gene names and a column of synonym gene names was saved. This table is the discoverable gene set.

Note: If the user decides to update the discoverable gene set using more recent downloads of HMDB and UniProt, the workflow will allow them to do so in module 3. More detailed instructions on updating the discoverable gene set are provided in Appendix D of the tutorial supplied in Supplementary Materials 1.

***Module 2 – Identify the metabolite-interacting proteins and their human genes***

This module finds proteins that are known to interact with metabolites. It searches metabolite HMDB codes (obtained from the “name_map” file) on the online HMDB database (3). For each metabolite, it grabs the name and UniProt id of interacting proteins as well as the corresponding gene names.

To ensure gene names are consistent between the metabolite genes (output in module 2) and the disease genes (output in module 3), gene names are standardized using the discoverable gene set supplied in “discoverableGenes_akaAllGenesInHMDB.RData”. This file is also used in the generation of the full GWAS network that is used as input in module 3. This ensures the same background for both the metabolite gene set and the trait/disease gene set. When standardizing gene names, if an identified metabolite-interacting protein gene is present as a primary gene in the discoverable gene set, it is kept unaltered. If a gene is present as a synonym gene in the discoverable gene set, it will be replaced by the primary gene name. If a protein’s gene is not present in the discoverable gene set or it is a synonym mapping to more than 3 primary gene names, the protein is removed. After standardizing gene names, the final gene set is the metabolite-interacting gene set.

***Module 3 –*** ***Trait/disease-related GWAS network preparation from the GWAS Catalog***

This module uses a network representation of the GWAS Catalog (4) and conducts a keyterm search operation to accumulate trait or disease-specific genes. As transforming the GWAS Catalog into a network is time-intensive, we supplied the full discoverable GWAS-network in “GWASnetwork_discoverableGenesOnly.RData” which users can input into module 3 (Fig. 1). Module 3 will then filter the network based on the keyterm input by the user to create a trait/disease-related GWAS subnetwork and gene set. Creation of this subnetwork and the keyterm search operation are described below.

*Formation of the full, discoverable GWAS network:*

The GWAS Catalog was downloaded as a table of associations in a tsv file. The tsv file contained rows representing studies, the traits they were testing, and the genes that were found to be associated with the traits. If there were no mapped genes, the row was removed. To create nodes, unique combinations of a study ID and a trait were generated. This was necessary as some studies investigated more than one trait. For each node, a list of genes found associated with the trait, are stored as a node attribute. Using the discoverable genes set (from discoverableGenes_akaAllGenesInHMDB.RData), the genes are standardized to their primary names and non-discoverable genes are filtered out (Fig. 2).

Edges between nodes are used to reflect the genetic overlap between the study-trait nodes. Overlap is quantified by Jaccard coefficients (centered) and p-values of the coefficients. Two matrices are created, one storing the coefficients and one storing the p-values. In each matrix, each node is represented by one column and one row. These matrices are supplied as R objects in “GWASnetwork_discoverableGenesOnly.RData” and represent the full discoverable GWAS-network (Fig.2).


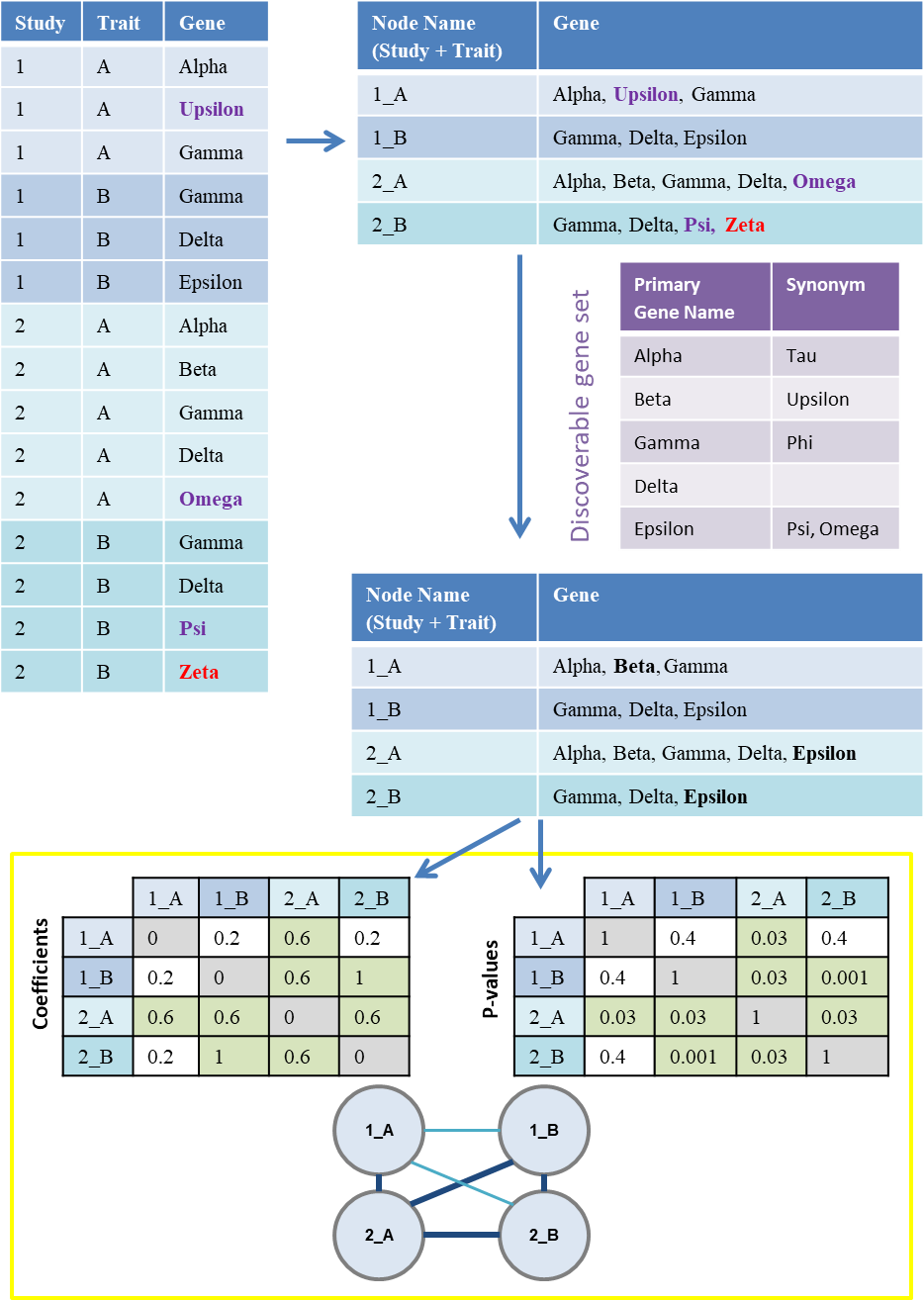


**Figure 2 *(previous page)*. Full discoverable network creation.** The downloaded GWAS Catalog is a table (top left) with studies, traits and genes. The table is collapsed into unique study+trait combinations for which genes are accumulated (top right). Gene names are then filtered to remove non-discoverable genes (red text) and to standardize synonyms (purple text) to primary names (black text). The discoverable gene set (purple table) is used to accomplish this. Each study+trait from the collapsed table is a node in the network. Edges in the network are represented by genetic overlap of nodes. Genetic overlap is quantified between all pairs of nodes by a Jaccard coefficient (left matrix) and a Jaccard p-value (right matrix). Nodes do not connect to themselves, therefore diagonals in the matrix (grey cells) for these coefficients and p-values are set to 0 and1 respectively. These two matrices represent the full, discoverable network structure. Edges with smaller coefficients and larger p-values (white cells in matrices; thin, light blue edges in network) are less likely to pass user thresholds while edges with larger coefficients and smaller p-values (green cells in matrices; dark, thick edges in network) are more likely to pass user thresholds.

Note: The supplied GWAS network was created using the GWAS Catalog from December 2019 (“All associations v1.0” file downloaded from <https://www.ebi.ac.uk/gwas/docs/file-downloads>). As the GWAS network and the discoverable gene set are built using the GWAS Catalog and UniProt/HMDB databases respectively, all of which will change over time as new discoveries are made, the workflow provides an option to update both objects during module 3. Note that the updates are slow and should therefore not be done regularly. Additionally, we will update these objects and RData files periodically and upload them to GitHub (after acceptance of this paper). The GitHub address will be appended in the main manuscript later.

*Searching by a keyterm:*

Module 3 requires the adjacency matrices representing the network structure of the GWAS Catalog. When given the adjacency matrices, the module applies user provided thresholds on the Jaccard coefficient and FDR-corrected p-values to determine which nodes have links between them in the network (Fig.3). A conservative threshold for Jaccard coefficients would be 0.5 while a more moderate threshold would be 0.25, according to documentation for Cytoscape's enrichment map (<https://enrichmentmap.readthedocs.io/en/latest/Parameters.html>).

Next, the module requires a search term. The module selects all nodes containing that search term in their name (i.e., the study title or the trait being looked at) and also selects all the 1^st^ order neighbors of those nodes as being likely relevant to the key term due to a significant overlap in genes (Fig.3). For example, if searching diabetes, studies about insulin may be of interest. Note that capitalization is ignored during matching of keyterms. Unselected nodes are removed from the network. The remaining network is known as the trait/disease-related GWAS subnetwork and the genes present in it are the trait/disease-related genes.

Note: If a disease gene set for more than one key term is desired, this module should be run once for each term, and the disease gene sets for the terms should be merged together before running module 4.


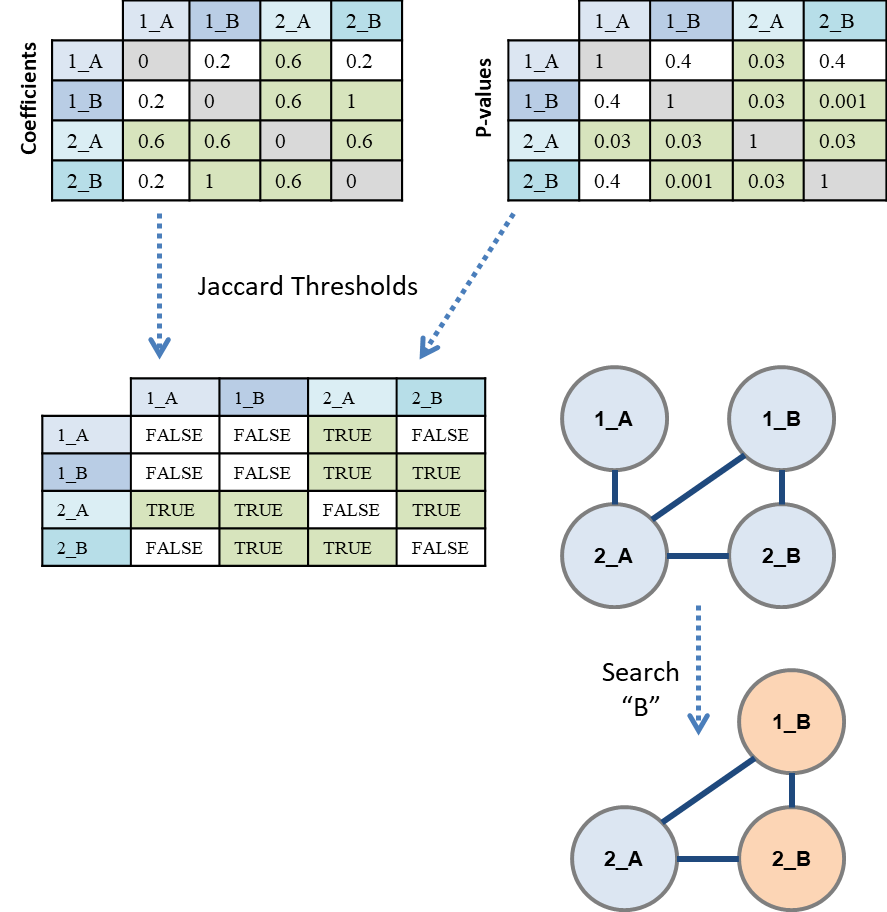


**Figure 3. Filtering the full GWAS network to obtain a trait/disease network.** The matrices representing the full GWAS network (top two matrices) are input into the workflow. The user supplies thresholds for the Jaccard coefficients and associated p-values. In this example the coefficient and p-value thresholds used were ≥ 0.5 and ≤ 0.05 respectively (green cells). Edges where both the coefficient and p-value pass the threshold are kept in the network (a matrix is created with True and False representing whether edges are present of not in the network). Next, the user supplies a keyterm, for example “B”. Nodes with B in their study title or trait are selected (highlighted in orange) and 1^st^-order neighbors are also kept. All other nodes are removed. This filtered network is the trait/disease network.

Although not discussed in the main manuscript, this trait/disease-related GWAS network can then be graphed and clustered. Provided there is more than one node in the network, the workflow will present the user with this option. Clustering is done with igraph’s (7) “cluster_fast_greedy” algorithm and uses negative log-transformed p-values as weights. Clusters are named after the most common words in the node names. The top 5 words are used. If there is a tie between words, the tie is broken by alphabetical order. Results include word counts used when naming the clusters and these should be verified for ties (Fig. 3). For details on other results output by this module, see Appendix F of the tutorial document.

| 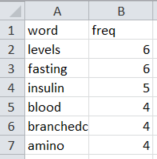 | **Figure 3: Cluster names should be verified before interpretation.** Word counts used to name clusters are organized in sheets of an excel worksheet file. In this example, naming the cluster after the first five words results in the name “levels_fasting_insulin_blood_branchedchain”. Amino is just as frequent as blood and branchedchain but was not used in the name as it is listed 6^th^. |
| --- | --- |

***Module 4 – Over-representation analysis for genetic predisposition***

This module tests whether metabolite genes are over-represented in disease genes using a hypergeometric test. A hypergeometric test determines if the number of successes in a draw is different than expected by chance when drawing from a background with a fixed number of successes and failures. Here, the background is the discoverable gene set and the successes are the disease genes (from module 3). The metabolite gene set (from module 2) can be considered the draw and the observed successes in the draw are the overlapping genes between the metabolite and disease gene sets. The module returns the upper-tail p-value.

REFERENCES

1. Xia, J., Psychogios, N., Young, N. & Wishart, D. S. MetaboAnalyst: A web server for metabolomic data analysis and interpretation. *Nucleic Acids Res.* **37**, 652–660 (2009).
2. Ogata, H. *et al.* KEGG: Kyoto encyclopedia of genes and genomes. *Nucleic Acids Res.* **27**, 29–34 (1999).
3. Wishart, D. S. *et al.* HMDB 4.0: The human metabolome database for 2018. *Nucleic Acids Res.* **46**, D608–D617 (2018).
4. Buniello, A. *et al.* The NHGRI-EBI GWAS Catalog of published genome-wide association studies, targeted arrays and summary statistics 2019. *Nucleic Acids Res.* **47**, D1005–D1012 (2019).
5. Chung, N. C., Miasojedow, B. Z., Startek, M. & Gambin, A. Jaccard/Tanimoto similarity test and estimation methods for biological presence-absence data. *BMC Bioinformatics* **20**, 1–11 (2019).
6. Neo Christopher Chung, Blazej Miasojedow, Michal Startek and Anna Gambin (2018). jaccard: Test Similarity Between Binary Data using Jaccard/Tanimoto Coefficients. R package version 0.1.0. https://CRAN.R-project.org/package=jaccard
7. CSARDI, G. & NEPUSZ, T. The igraph software package for complex network research. *InterJournal* **Complex Sy**, 1695 (2006).
