# Supplementary material for: metGWAS 1.0: an R workflow for network-driven over-representation analysis between independent metabolomic and meta-genome-wide association studies
Source: Bioinformatics. 2023 Aug 23;39(9):btad523. doi: 10.1093/bioinformatics/btad523 (PMC10491949; doi:10.1093/bioinformatics/btad523)
Supplement: btad523_Supplementary_Data [file btad523_supplementary_data.zip › Supplementary Material 1/metGWAS tutorial.docx]

**Tutorial on how to run metGWAS 1.0 (Network driven over-representation analysis between independent metabolomic and meta-genome wide association studies identifies gene-metabolite- relationships to phenotype).**

INTRODUCTION

Genetic predispositions can influence the dysregulation of metabolites in diseases. Paired metabolomic and genotyping studies can determine which variants are responsible for particular metabolic dysregulation, but most datasets are not paired. The metGWAS 1.0 platform allows us to identify possible genetic predispositions (disease-risk-allele-containing gene loci) from standalone metabolomics data. This tutorial will describe how to identify a metabolite gene set and a trait/disease gene set, and test for significant overlap between these two gene sets. Conceptual details of the workflow, which is split into four modules, are found in the associated publication and the technical document in Supplementary Material 1.

We provide example data sets as a vignette. The GWAS catalog (as of Dec 2019) is supplied as a network with all Jaccard coefficients and p-values precalculated. The set of metabolites is from a study on cardiovascular disease (CVD) (*1*). The metabolites are the set of all lipids that met Tabassum *et al.*’s study-wide significance level (p < 1.5 x 10^-9^) and were provided in the folder “..\Supplementary Material 2\Tabassum\PathwayAnalysisResults_fromPreviousSubmission”. The metabolite data set was assessed by MetaboAnalyst 5.0’s Pathway Analysis module (*2,3*) to identify KEGG pathways. MetaboAnalyst CSV formatted output files– ‘pathway_results’ and ‘name_map’ – are supplied as the starting material for module 1 of the metGWAS 1.0 platform (details provided in this tutorial).

metGWAS 1.0 was tested using R version 3.6.3 and RStudio version 1.1.456 (*4,5*), although we do not believe other versions of RStudio would pose a problem. R can be downloaded at <https://cran.r-project.org/> and RStudio can be downloaded at <https://rstudio.com/products/rstudio/download/#download>. The workflow is interactive to support users without previous R experience, and a short introduction to RStudio is provided in **Appendix A**. Running the workflow will display output in the console, where the user can input information the workflow prompts them for. In this tutorial, font color denotes:

Input/output in the **console** is represented by a light grey background surrounded by a box

(with ... on the darker grey background representing any skipped output.)

Dark blue text represents output from the workflow,

orange represents input the user provides,

and red represents additional details output by RStudio.

**SET-UP**

*Overview of Steps*

- Step 1: Download workflow, supporting objects, and data for this tutorial.
- Step 2: Install qvalues and bioconductor.
- Step 3: Set-up RSelenium.

Note: These steps only need to be done once before running the workflow for the first time. Subsequent runs of the workflow at later times/dates do not require this set-up.

*Files Required*

- Step 1 – Download workflow, supporting objects, and data for this tutorial.

The supplied data for this tutorial is a set of metabolites dysregulated in CVD. It originated from *Tabassum, R. et al.* (*1*) and can be found in Supplementary Material 2 (sub-folders “Dataset 2 Tabassum” and MetaboAnalyst_PathwayAnalysis results). Users can replace this data with their testing dataset during their actual operation.

The workflow is run by the R script “metGWAS_2021_12_01.R”. This file can be found in our supplied Supplementary Material 1 associated with the publication. Two other R scripts are required to help set-up the workflow. These are “qvalue and bioconductor install.R” and “RSelenium Driver SetUp.R” and both can be found in our supplied Supplementary Material 1. Additionally, the workflow uses certain data objects that have been stored in two RData files (Fig. 1B):

- Discoverable gene set for standardizing gene names and maintaining appropriate background:
  - discoverableGenes_akaAllGenesInHMDB_2021_10_14.RData
- Full discoverable GWAS network (with appropriate background for comparison to metabolite genes):
  - GWASnetwork_discoverableGenesOnly_2021_10_14.Rdata

These two files are also found in our Supplementary Material 1 in the folder called “objects”. They can be updated by the workflow if desired (in module 3).

| 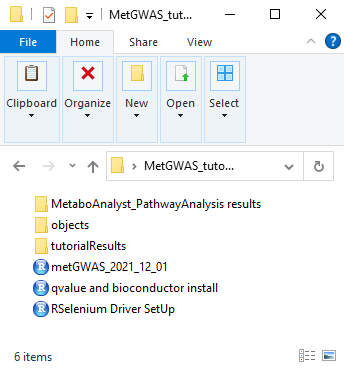  A | **Figure 1: Suggested file organization.** Keeping data together as much as possible (especially in the training phase) will make it easier to use the workflow and follow the tutorial.  To follow this tutorial, we suggest creating a main folder (A) with the workflow script “metGWAS_2021_12_01” and the other 2 scripts used in set-up, as well as sub-folders “objects” and “MetaboAnalyst_PathwayAnalysis results”. The latter can be downloaded from our Supplementary Material 2 (sub-folder “Dataset 2 Tabassum”), while the other files can be downloaded from Supplementary Material 1. We have also created sub-folder “tutorialResults” to store the results of the workflow when it is run.  (B) The 2 RData files containing objects the workflow uses are kept in the “objects” sub-folder.  (C) The sub-folder “MetaboAnalyst_PathwayAnalysis results” contains csv file “metabolites_fromTabassum” which lists the HMDB ids used as input in MetaboAnalyst and csv files “name_map” and “pathway_results” which are the results from MetaboAnalyst. |
| --- | --- |
| 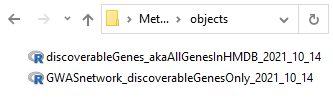  B |  |
| 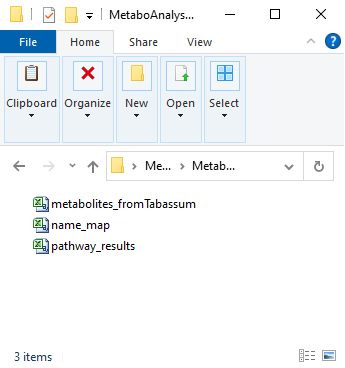  C |  |

*R Packages Required*

The workflow uses the following R libraries:

- DT (version 0.13)
- igraph (version 1.2.5)
- jaccard (version 0.1.0)
- openxlsx (version 4.1.4)
- RSelenium (version 1.7.7)
- rvest (version 0.3.5)
- scales (version 1.1.0)
- shiny (version 1.4.0.2)
- tidyverse (version 1.3.0)
- tm (version 0.7-7)
- wordcloud (version 2.6)
- XML (version 3.99-0.3)

To install the above packages first complete steps 2 and 3 (described below). This will set up the automatic installation when the R-script “metGWAS_2021_12_01” is run (by clicking the source button of R-studio).

- Step 2 – Install qvalues and bioconductor.

The package *qvalue* (version 2.18.0) is required to install the Jaccard package listed above. For R 3.6.3, qvalue is not available on CRAN and must be installed via Bioconductor version 3.10 (6*,7*), before running the workflow for the first time. A script to install these packages is supplied in our Supplementary Material 1 (named ‘qvalue and biconductor install.R’). To install both bioconductor and qvalue, open the script “qvalue and bioconductor install.R” in RStudio and click the source button (Fig. 2).

| 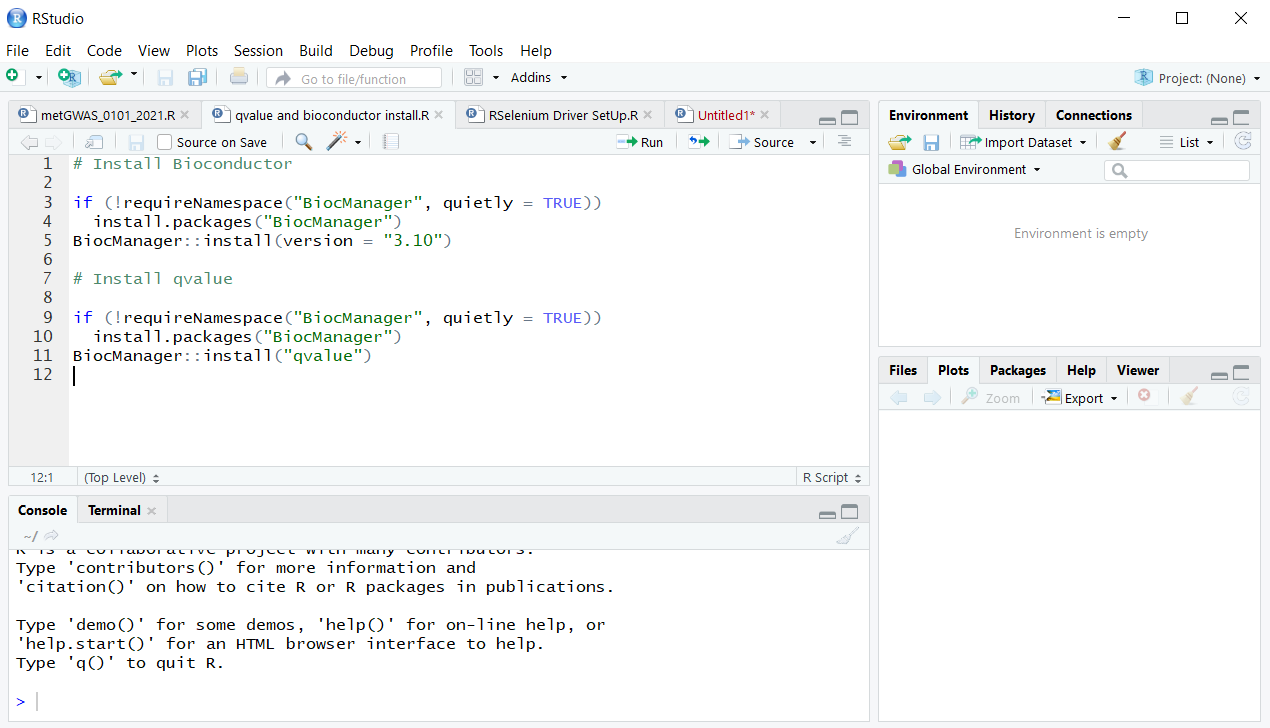 |
| --- |
| **Figure 2: Install bioconductor and qvalue.** Open the R file “qvalue and bioconductor install” in RStudio. Ensure you are on the tab with the same name. Click the source button. |

- Step 3: Set-up RSelenium.

The package *RSelenium* (*8*) is required before running the workflow for the first time. This is installed by opening the file “RSelenium Driver SetUp.R” within RStudio and clicking the source button (Fig. 3). When the script is finished, it will notify you to restart RStudio. Close RStudio and open it again. We are now ready to begin the workflow. If interested, a brief explanation of why this separate set-up is required is provided in the comments at the top of the script.

| 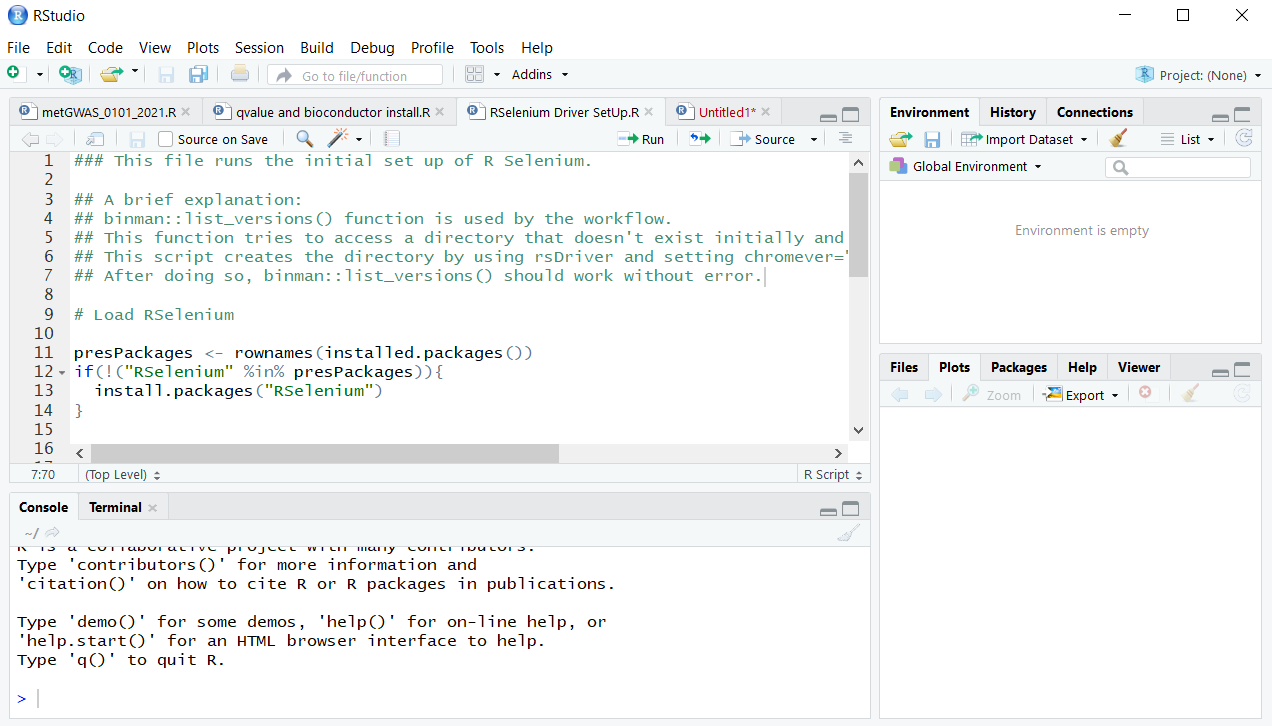 |
| --- |
| **Figure 3: Setting up RSelenium.** Ensure you are on the tab “RSelenium Driver SetUp.R”. Click the source button. When the console (green box) indicates the script is finished, restart RStudio. |

**STARTING THE WORKFLOW AFTER SET-UP**

*Overview of Steps*

- Step 1: Open metGWAS_2021_12_01.R and click source.
- Step 2: Notify workflow if R objects will be used as input.
- Step 3: Libraries are loaded.
- Step 4: Input chrome driver version.
- Step 5: Input where to save results.

*Starting the Workflow*

- Step 1 – Open metGWAS_2021_12_01.R and click source.

Open the script “metGWAS_2021_12_01.R” in RStudio. The workflow is started by pressing the **source** button (Fig. 4). This will run the R script and the workflow will prompt the user for input in the console. See **Appendix A** for a brief introduction to RStudio.

| 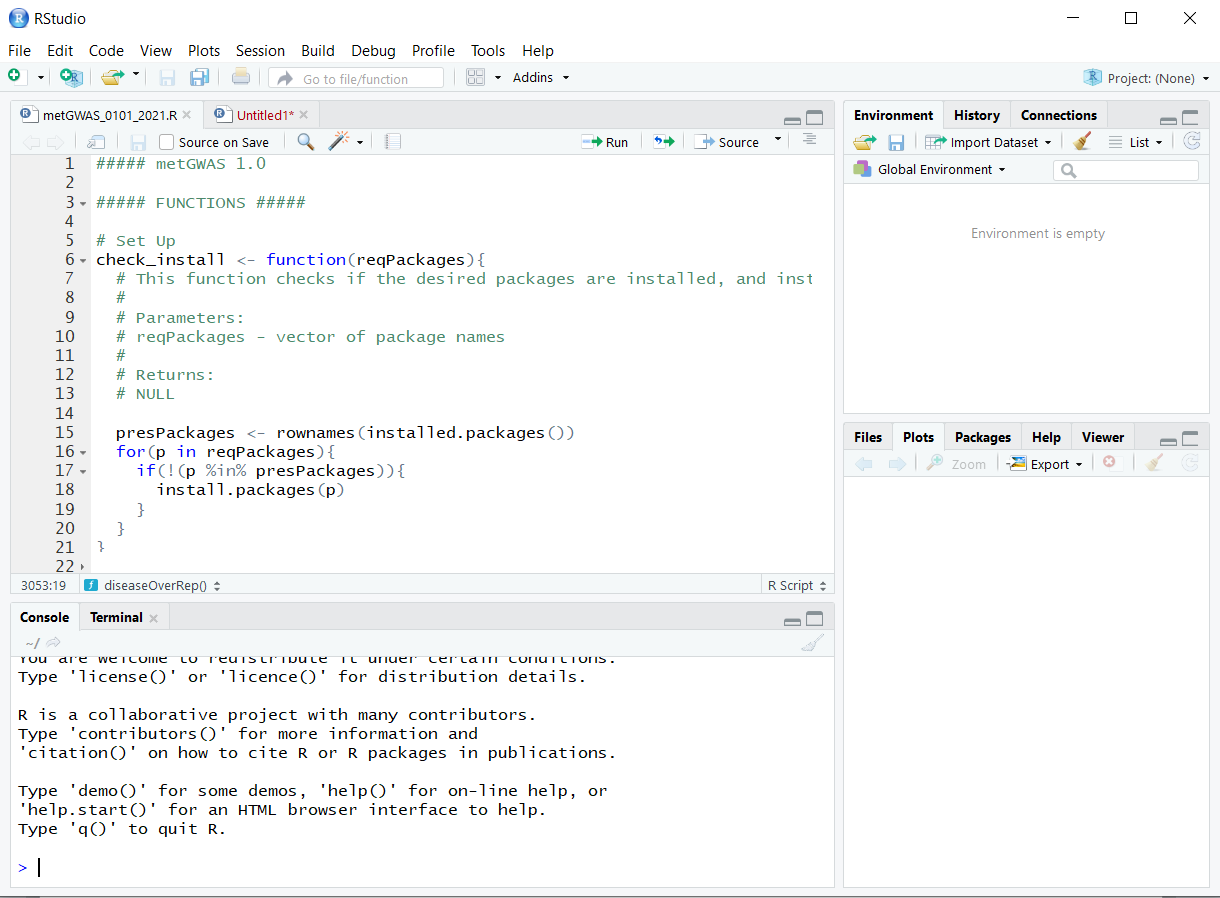 |
| --- |
| **Figure 4: Starting the workflow.** Open script “metGWAS_2021_12_01.R”. With RStudio open, this can be done by going to File (red box) > Open File and then selecting the script. Click source (red circle).The workflow will begin to display output in the console (green box). |

- Step 2 – Notify workflow if R objects will be used as input.

The first message displayed will remind us that if the data we are analyzing is stored in R objects, the objects will need to be present in the R environment before starting the workflow. Our data is stored in csv files (not in R objects), therefore, we will input “n” (for “no”). We recommend using csv files, especially if you are unfamiliar with R.

Users can see the following statement in the console:

Welcome.

This workflow requires data input. Generally, the user is given the choice between inputting R objects or csv files. If inputting R objects, these objects must be loaded into the environment before starting the workflow.

For more details see the walkthrough tutorial.

Do you intend to use R objects as input at any point (y or n)?

n

Note that the R objects the workflow warns about do not include the objects in the three RData files deposited with the workflow (see Set-Up: Files Required), as these are reference datasets, not the data of interest. We will be prompted for these three files later in the workflow. For more information on inputting R objects instead of csv files, please see **Appendix B**.

- Step 3 – Libraries are loaded.

Next, the workflow installs any missing packages. If we wanted to manually install the packages, we could quit the workflow (by entering “q”) at this point. However, we will allow the workflow to do this for us (by entering “c”). Importantly, we have already installed the packages *qvalue* and *RSelenium* ourselves (see Set-Up: Packages).

Once packages are installed, the libraries are loaded into the environment. Loading libraries will result in output on the console that we can ignore. The workflow will notify us once the libraries have been loaded.

This workflow requires the following packages to be installed:

XML

RSelenium

rvest

tidyverse

igraph

jaccard

tm

wordcloud

scales

shiny

DT

openxlsx

If you would like to manually install these packages, please enter quit, install the packages, and restart (source) this workflow.

If you would like to allow the workflow to install any missing packages (or you've already installed the packages yourself), please enter continue.

Quit = q

Continue = c

Quit or continue:

c

Packages present/installed.

Loading libraries...

(Do not worry if output shows up on the console.)

Loading required package: xml2

-- **Attaching packages** --------------------------------------- tidyverse 1.3.0 --

v ggplot2 3.3.0 v purrr 0.3.3

...

Libraries have been loaded.

- Step 4 – Input chrome driver version.

Next, the workflow will ask what version the driver for our Google Chrome browser is. This can be found by opening the menu in chrome, going to “Help” and then “About Google Chrome” (Fig. 5).

| 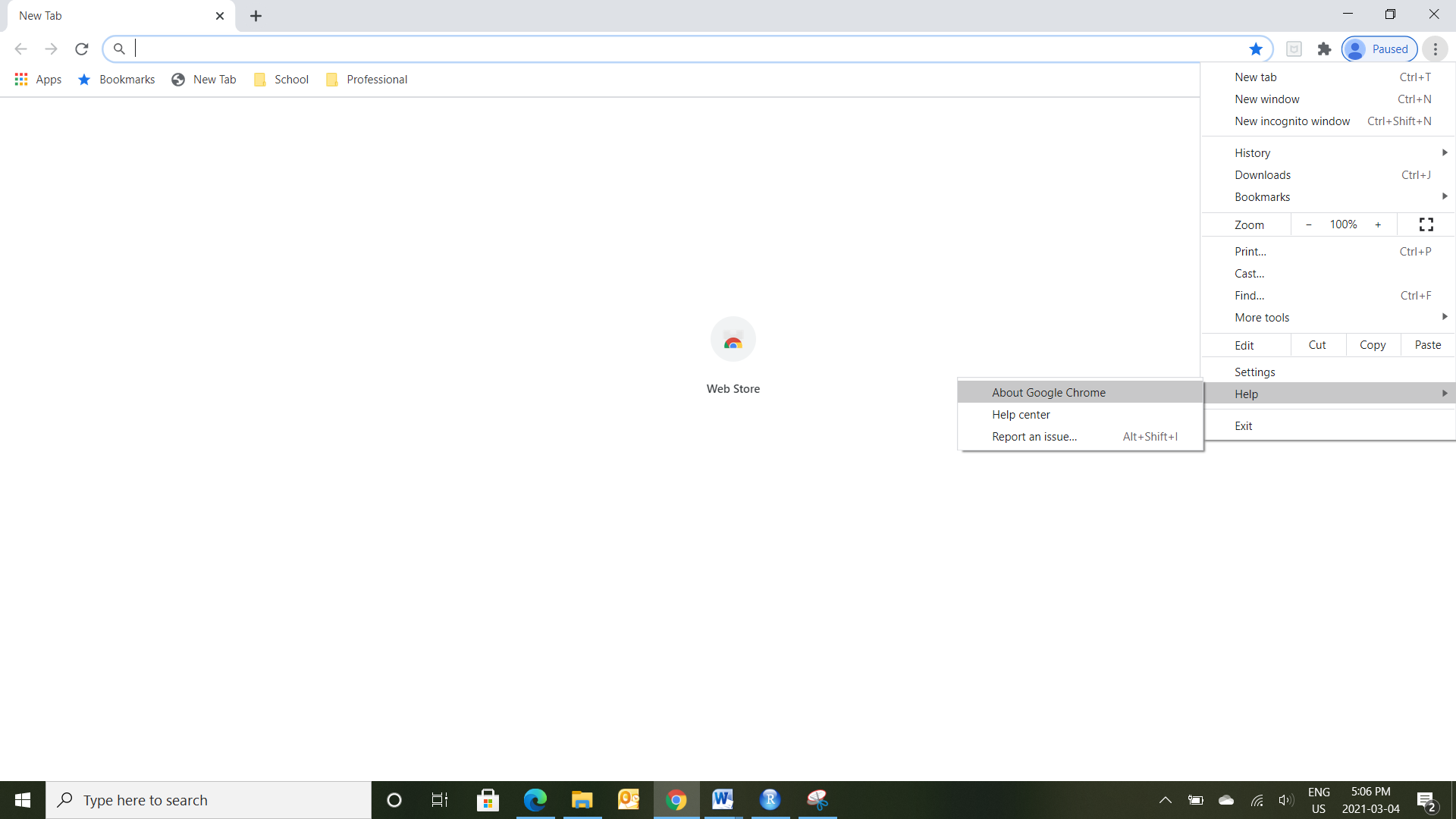  **A** | 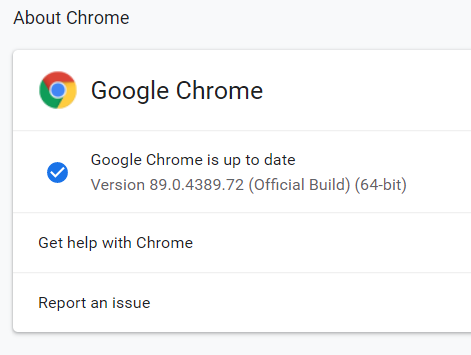  **B** |
| --- | --- |
|  | **Figure 5: Finding your Chrome driver version.** (A) Go to Help > About Google Chrome. (B) Information about your version will be displayed. |

Input the version number listed by the workflow that best matches your version (the right-most numbers may not be an exact match to your version). In this example, chrome is version 89.0.4389.72 but the input entered is 89.0.4389.23 as that is the best match.

Some modules in this workflow will need to interact with your chrome browser.

Please check what version your chrome driver is (and press enter to continue).

The following are different versions of chrome drivers that this workflow can use:

For win32:

81.0.4044.138

83.0.4103.14

83.0.4103.39

84.0.4147.30

85.0.4183.38

85.0.4183.83

85.0.4183.87

86.0.4240.22

87.0.4280.20

87.0.4280.88

88.0.4324.27

88.0.4324.96

89.0.4389.23

Please select a driver from the above list that most closely matches your current chrome driver. The left numbers in each version are more important, the numbers after later decimals do not need to match exactly.

If none of these work, you may need to update your chrome driver first.

Please enter the matching driver from the above list (enter the full version even if it doesn't match exactly:

89.0.4389.23

- Step 5 – Input where to save results.

The workflow will now ask us for a directory and folder where we would like to save the results. Throughout the workflow, there are prompts to save results at the end of each module. The location you provide is where all results will be saved. Note, pressing enter without providing a path will prevent prompts to save results. The results will exist as R objects in the work environment and can be manually saved after completing the workflow.

In the console, copy and paste the directory and folder for saving results (Fig. 6). If the file path has the prefix “file:///”, it will need to be deleted.

| 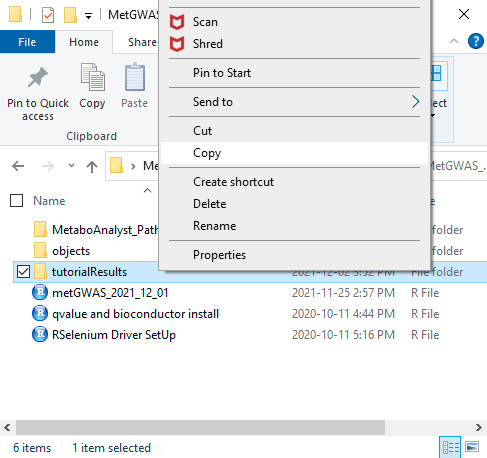  **A** | 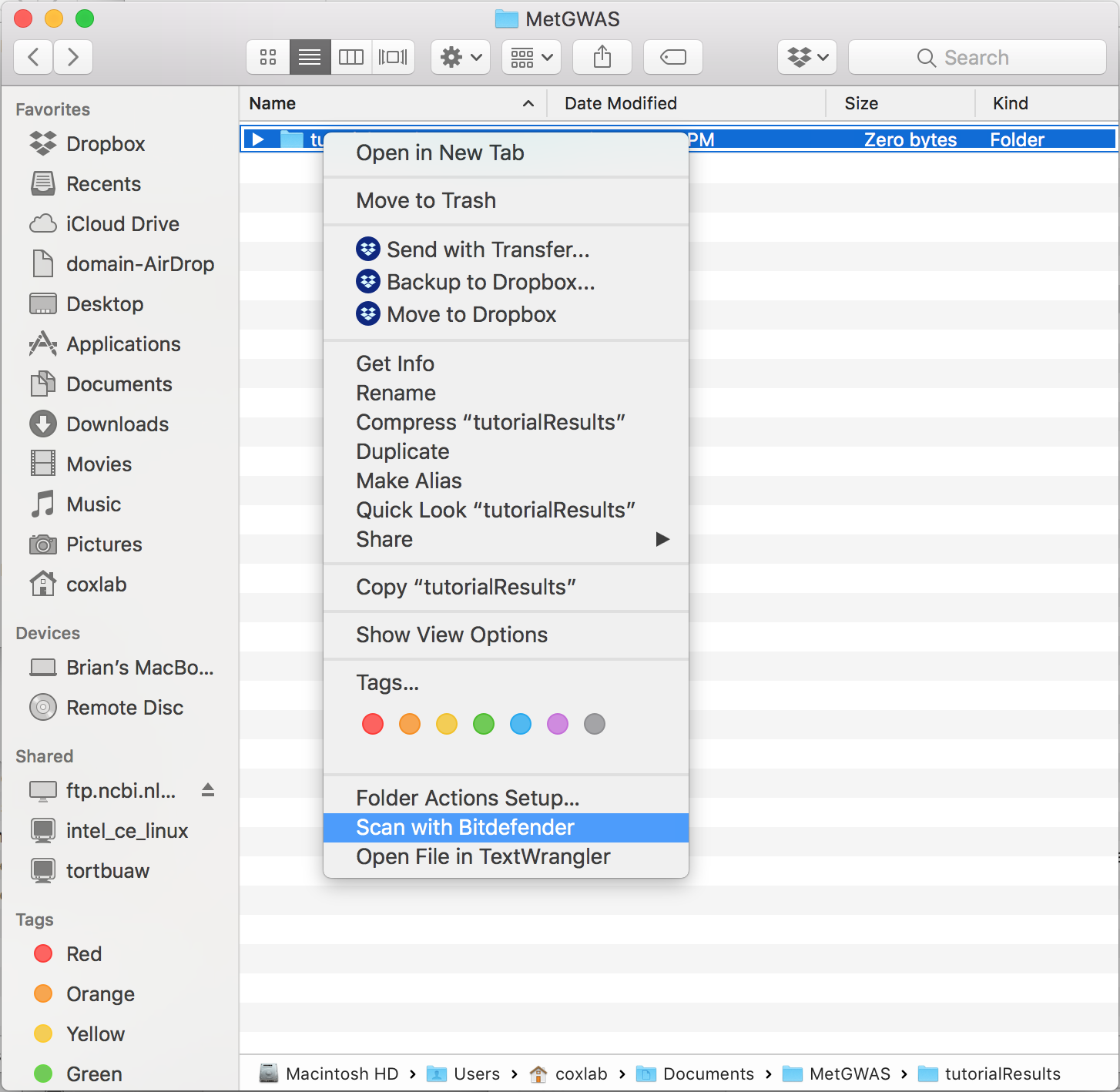  **B** |
| --- | --- |
| **Figure 6: Obtaining a path for a directory.** (A) In Microsoft Windows: To copy the pathway to this directory and folder into the console, in file explorer (outside of RStudio), right-click the folder and select copy as shown. Then in RStudio’s console, right-click and select paste. (B) In OSX on an Apple computer: Open the finder and right click the folder of interest (*control + click*). Next, while the menu is open, press and hold the *alt/option* key. This will reveal another set of menu options. The option **Copy “tutorialResults”** will now read **Copy “tutorialResults” as Pathname.** Continuing to hold the *alt/option* key select the copy as pathname option. Then you can use *control + c* to paste the pathname. | |

Note that a windows pathnames uses the back slash “\” to separate the folder and file names and OSX uses the forward slash “/”. Although R expects file paths to contain forward slashes, metGWAS is programmed to accept both forward and backslashes as folder separators.

If you would like the workflow to automatically save its results, please enter a file path.

If you would like to save results yourself after the workflow is complete, press enter without supplying a file path.

File path:

D:\JohnDoe\MetGWAS_tutorial\tutorialResults

The path you entered was:

D:\JohnDoe\metGWAS_tutorial\tutorialResults

Proceed (y or n)?

y

Workflow beginning.

After confirming the file path for saving results, the workflow is ready to begin running modules. Each module will prompt to start. This allows stopping at any point of the workflow.

**IDENTIFYING A METABOLITE GENE SET**

*Overview of Steps*

Module 1 - *Identification and annotation of the corresponding metabolites for the selected over-represented KEGG pathways*

- Step 1: Describe format of pathway analysis results from MetaboAnalyst.
- Step 2: Select threshold to filter pathways by significance (i.e., FDR) and impactfulness (i.e., pathway impact).
- Step 3: Input pathway analysis results (i.e., pathway-results and name_map).
- Step 4: KEGG will be searched to map metabolites to pathways.
- Step 5: Save results.

Module 2 - *Identify the metabolite-interacting proteins and their human genes*

- Step 6: State whether module 2 is run in the same source as module 1.
- Step 7: HMDB will be searched for proteins that interact with metabolites of interest and their corresponding genes will be identified.
- Step 8: Input the discoverable gene set for standardizing gene names to primary gene names and finalize the metabolite gene set.
- Step 9: Save results.

*Descriptions of Steps for identifying a metabolite gene set*

*Module 1 – Identification and annotation of the corresponding metabolites for the selected over-represented KEGG pathways*

Module 1 will take outputs (i.e., ‘pathway_results’ and ‘name_map’ CSV files) from MetaboAnalyst 5.0’s Pathway Analysis module. Provided is example data from *Tabassum, R. et al.* (*1*) processed by MetaboAnalyst 5.0’s Pathway Analysis module. The results are provided as csv files in Supplementary Material 2 (sub-folder “Dataset 2 Tabassum”). These are in the sub-folder “MetaboAnalyst_PathwayAnalysis results” of Fig. 1.

- Step 1 – Describe format of pathway analysis results from MetaboAnalyst.

Module 1 of metGWAS 1.0 can accommodate several file formats, including MetaboAnalyst quantitative or qualitative results as either csv files or R objects from the MetaboAnalyst R package. The provided data are csv files of qualitative data from MetaboAnalyst. Select the appropriate options.

Do you wish to run the Pathways of Over-Representation module (y or n)?

y

This module uses results from MetaboAnalyst's pathway analysis module (see manual for more details).

Was your input into MetaboAnalyst qualitative (a list of differentially expressed metabolites) or quantitative (a concentration table)?

1 - qualitative

2 - quantitative

Enter a number:

1

What format are your results?

1 - R object created using package MetaboAnalystR.

2 - Csv files created and downloaded from MetaboAnalyst online.

Enter a number:

2

- Step 2 – Select threshold to filter pathways by significance and impactfulness.

Next, select an option to filter pathways based on significance and/or impact. Significance is defined by an FDR-adjusted p-value and impact is defined by a pathway impact factor, both of which are calculated and outputted by MetaboAnalyst. To apply the filter, enter a value between 0 and 1. Pathways with FDR-adjusted p-value less than or equal to the entered value and impact greater than or equal to the entered value will be kept. For the tutorial, apply FDR p-value threshold of 0.05. Skip the impact filter by entering 0.

What significance threshold do you want to filter the FDR adjusted p values by?

0.05

What significance threshold do you want to filter the impact factor by?

0

- Step 3 – Input pathway analysis results.

Next, enter the file path and name of the MetaboAnalyst pathway results and the accompanying name map. When downloaded from MetaboAnalyst, these are automatically named “pathway_results.csv” and “name_map.csv” (Fig. 7). If the file path has the prefix “file:///”, it will need to be deleted.

| 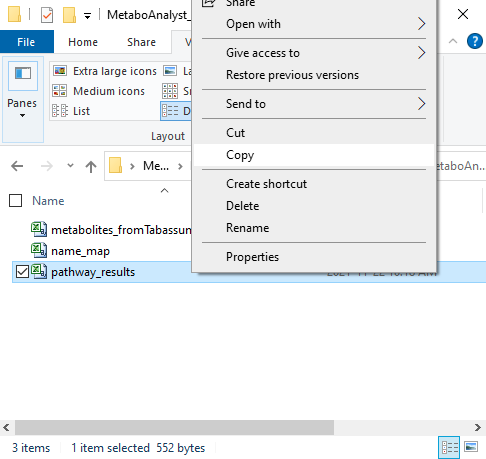  **A** | 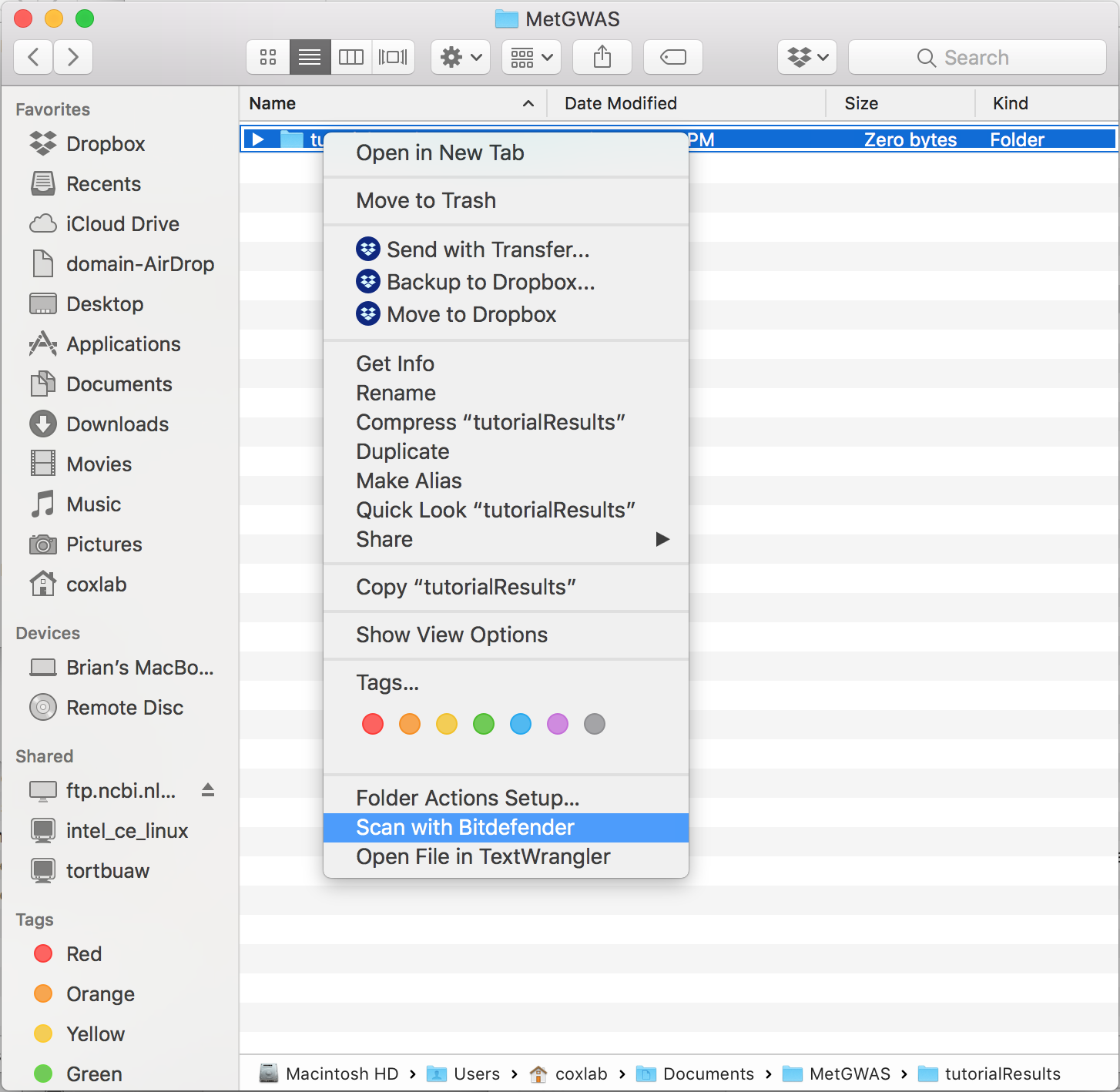  **B** |
| --- | --- |
| **Figure 7: Obtaining a pathway for a file.** (A) In Microsoft Windows: To copy a pathway to this file, find the file in file explorer (outside of RStudio), right-click on it, and select copy. Then, in RStudio’s console, right-click and select paste. (B) In Mac OSX: Open the finder and right click the file of interest (*control + click*). Next while the menu is open press and hold the *alt/option* key. This will reveal another set of menu options. The option **Copy “pathway_results”** will now read **Copy “pathway_results” as Pathname.** Continuing to hold the *alt/option* key select the copy as pathname option. Then you can use *control + c* to paste the pathname. | |

Using csv file...Please enter a file path to your pathway analysis results table:

D:/JohnDoe/MetGWAS_tutorial/MA_PathwayAna_Negative/pathway_results.csv

Please enter a file path to the name map generated by pathway analysis:

D:/JohnDoe/MetGWAS_tutorial/MA_PathwayAna_Negative/name_map.csv

- Step 4 – KEGG will be searched to map metabolites to pathways

Next, the workflow uses KEGG (*9*) to map which of the input metabolites belong to which of the enriched pathways. This requires an internet connection. A new chrome window will open to automatically search KEGG (Fig. 8). Do not work on or close the chrome window (minimizing the window is fine). Any other browser windows opened can continue to be used without interfering. If the chrome browser appears to be stuck, minimize it and check the RStudio console for output. If the console says there has been an error, you will need to close the chrome browser and restart RStudio (close and reopen) before you restart the workflow.

Chrome will pop up and the workflow will automatically control it.

DO NOT INTERFER WITH THE BROWSER.

Press enter to continue...

checking Selenium Server versions:

BEGIN: PREDOWNLOAD

...

| 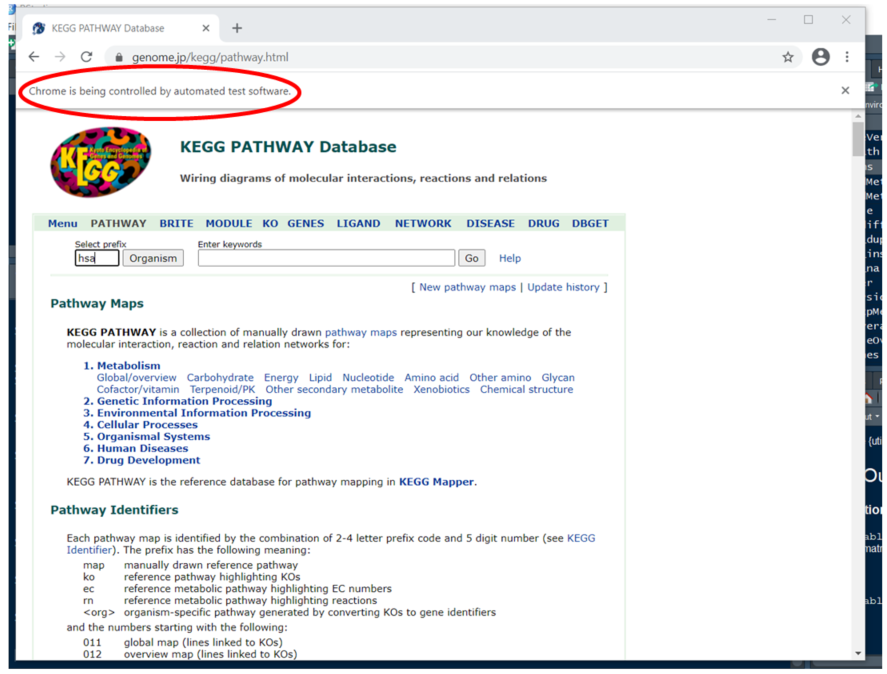 |
| --- |
| **Figure 8: Example of chrome browser that pops up due to the workflow.** There will be a notice that the browser is under control of automated software. The webpage in the browser window will automatically change as the workflow searches different pathways within KEGG. |

When all metabolites are mapped, the browser will close and the workflow will display metabolites that have more than one HMDB id (in this case, there are none, indicated by “0 rows”).

SUCCESS: The process "java.exe" with PID 14316 has been terminated.

The following metabolites found in the over-represented pathways had more than one HMDB id:

[1] metNames HMDBcode

<0 rows> (or 0-length row.names)

- Step 5 – Save results.

Module 1 is now completed and a prompt to save the result needs to be answered before proceeding to the next module. Saving options include as an R object or as a csv file.

Pathway Over-Representation Analysis module complete!

Do you wish to save the results of this module (y or n)?

y

How do you wish to save the results?

1 - as R objects in an RData file

2 - as csv files

3 - both

4 - neither

Enter a number:

3

Results were save in D:\JohnDoe\MetGWAS_tutorial\tutorialResults

In this example, we saved results in both formats (option “3’). The csv files output by the workflow (Fig. 9) should match those beginning with “mod1” in the “metGWAS results” sub-folder from Supplementary Material 2 (Dataset 2 Tabassum). The file we are most interested in is “**mod1_metaboliteNames_HMDBcodes**” as it contains all the metabolites from our input data that mapped to pathways passing our thresholds (applied in step 3). These are the metabolites that will be used in module 2. For more detailed descriptions of each file in the output, see **Appendix F**.

| 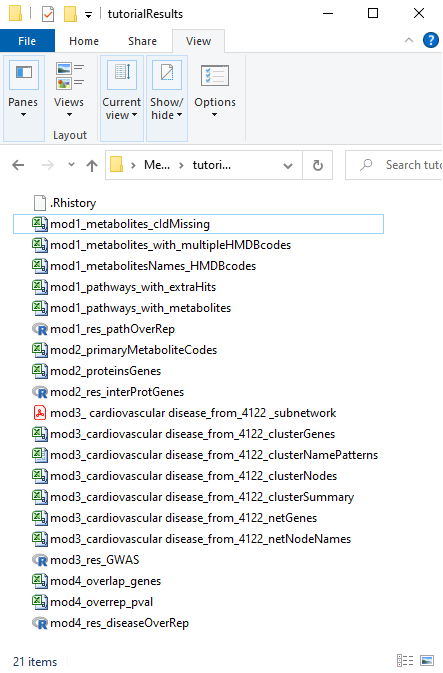  **A** | 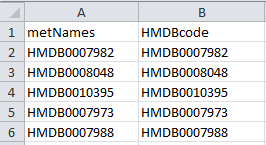  **B** |
| --- | --- |
|  | **Figure 9: Module output.** (A) All output from running the workflow is in folder “tutorialResults” and all output from module 1 starts with “mod1” (red box). Output from later modules will not be present until those modules are run. (B) The file “mod1_metaboliteNames_HMDBcodes” contains a column of metabolite names and their corresponding HMDB codes. In this case the metabolite names are HMDB codes as that was the format of the query entered into MetaboAnalyst. Only metabolites mapping to pathways passing thresholds applied in this module are present in this file. |

*Module 2 – Identify the metabolite-interacting proteins and their human genes*

- Step 6 – State whether module 2 is run in the same source as module 1.

Module 2 will identify proteins that interact with metabolites. Due to the modular design of metGWAS it is possible to start module 2 without running module 1. In this tutorial, we are running module 2 after module 1, without any interruption of the workflow, therefore we enter “y”.

Do you wish to run the “Interacting Proteins and Genes” module (y or n)?

y

Running Interacting Proteins and Genes module...

Are you running this module immediately after module 1 (Pathways of Over-Representation)? In other words, was this workflow uninterrupted (y or n)?

y

- Step 7 – HMDB will be searched for proteins that interact with metabolites of interest and their corresponding genes will be identified.

Next, the workflow will look up proteins that interact with the inputted metabolites and the corresponding genes using HMDB (*10*). Once again, the workflow will interact with the internet through the Chrome browser. As in module 1, a chrome browser will pop up. Do not close the browser, the workflow will do so when it is done, unless there is an error.

Chrome will pop up and the workflow will automatically control it.

DO NOT INTERFER WITH THE BROWSER.

Press enter to continue...

checking Selenium Server versions:

BEGIN: PREDOWNLOAD

BEGIN: DOWNLOAD

...

SUCCESS: The process "java.exe" with PID 14092 has been terminated.

- Step 8 – Input the discoverable gene set for standardizing gene names to primary gene names and finalize the metabolite gene set.

Once the workflow has identified the metabolite gene set (the previous step), it will filter it to contain only discoverable genes. These discoverable genes are the genes that code for all proteins known to interact with all metabolites in HMDB (not just the metabolites we are analyzing). It will also standardize gene names to primary gene names.

The workflow will ask us for the file path to the discoverable gene set. This is the RData file beginning with “discoverableGenes_akaAllGenesInHMDB” that came with this workflow (Fig. 14).

Please enter a file path to your discoverable gene set (an RData file):

D:/JohnDoe/MetGWAS_tutorial/objects/discoverableGenes_akaAllGenesInHMDB_2021_10_14.RData

- Step 9 – Save results.

When the workflow is finished standardizing gene names, the module is complete. As in module 1, there will be a prompt to save results.

Interacting Proteins and Genes module complete!

Do you wish to save the results of this module (y or n)?

y

How do you wish to save the results?

1 - as R objects in an RData file

2 - as csv files

3 - both

4 - neither

Enter a number:

3

Results were saved in D:\JohnDoe\MetGWAS_tutorial\tutorialResults

The csv files saved by the workflow should match those beginning with “mod2” in the “metGWAS results” sub-folder from Supplementary Material 2 (Dataset 2 Tabassum). The file “**mod2_proteinsGenes**” contains the proteins found for the metabolites of interest and their corresponding primary gene names (Fig. 10). For more detailed descriptions of each file in the output, see **Appendix F**.

| 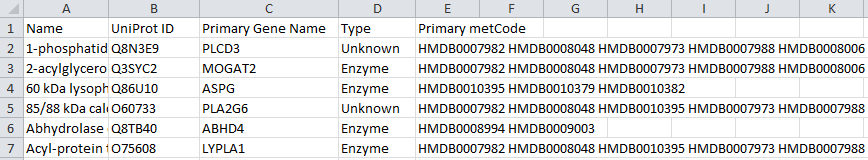 |
| --- |
| **Figure 10: Output “mod2_proteinsGenes”.** This file contains proteins (under “Name”) with their UniProt ids (under “UniProt ID”) and their “Primary Gene Name”, along with all the metabolites that were listed to interact with them in the online HMDB database (listed under “Primary metCode”). |

**IDENTIFYING A TRAIT/DISEASE GENE SET**

*Overview of Steps*

Module 3 - *Trait/disease-related GWAS network preparation from the GWAS Catalog*

- Step 1: Provide a file path to the existing GWAS Catalog network.
- Step 2: Apply thresholds to the network.
- Step 3: Explore search terms.
- Step 4: Select a final search term.
- Step 5: Graph the GWAS Catalog network.
- Step 6: Save results.

*Descriptions of Steps for identifying a trait/disease gene set*

*Module 3 – Trait/disease-related GWAS network preparation from the GWAS Catalog*

Module 3 uses a network representation of the GWAS Catalog (*11*) to collect genes relevant to a trait of interest. For conceptual details, please see the paper and the technical manual in Supplementary Material 1.

- Step 1 – Provide a file path to the existing GWAS Catalog network.

When running module 3, the workflow will need a matrix object representing the GWAS Catalog network. The required objects are provided in “GWASnetwork_discoverableGenesOnly_2021_10_14.RData”. This GWAS-network contains only discoverable genes or in other words, genes that code for any metabolite-interacting proteins (all proteins in HMDB). As we will be comparing the disease genes with the metabolite genes, it is important they have a matching background, which is why the GWAS-network provided only contains discoverable genes.

At this point the workflow presents an option to update the GWAS Catalog network. It is important to periodically update the GWAS network as the online database will change over time as new discoveries are made and deposited. Note that this is a time-intensive process. It will take upwards of 3 hours to convert the Catalog into a network. See **Appendix D** for more details. Here we do not update the network, and instead, load the existing network by providing a file path to the discoverable network (Fig. 11).

Do you wish to run the “GWAS Genes” module (y or n)?

y

Running GWAS Genes module...

Do you wish to update the full GWAS Catalog network (y or n)?

n

An existing full GWAS Catalog network will be loaded (for more details, see the user manual).

Please enter a file path to an .RData file with a network:

D:/JohnDoe/MetGWAS_tutorial/objects/GWASnetwork_discoverableGenesOnly_2021_10_14.RData

You have entered the following file path as your GWAS Catalog network:

D:/JohnDoe/MetGWAS_tutorial/objects/GWASnetwork_discoverableGenesOnly_2021_10_14.RData

Confirm (y or n)?

y

Loading...

| 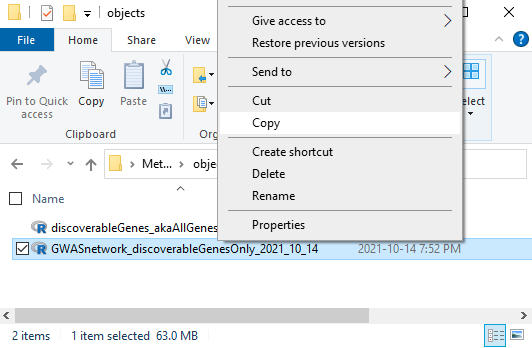  **A** | 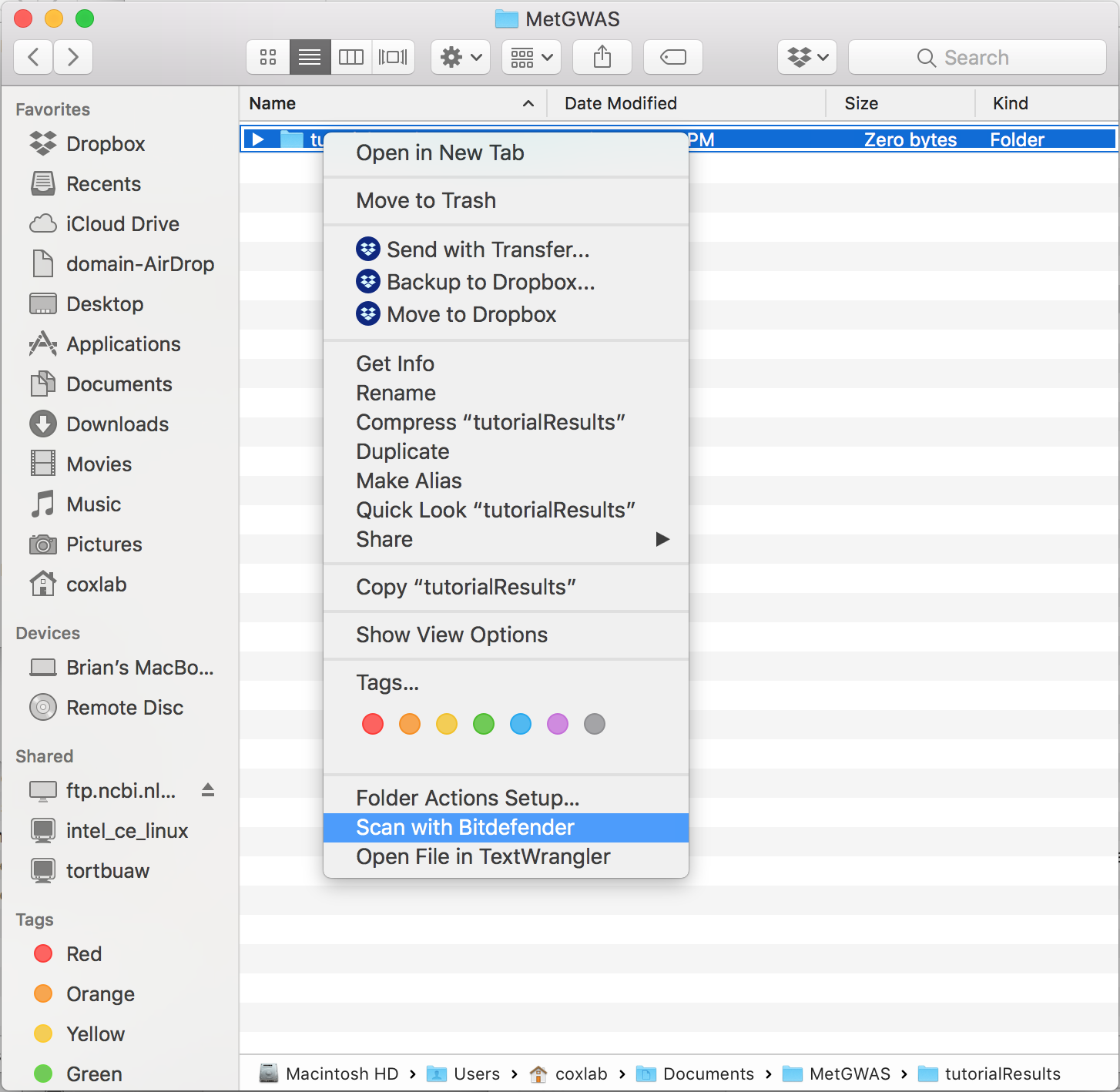  **B** |
| --- | --- |
| **Figure 11: Copy file path to the discoverable GWAS Catalog network. (A)** In Windows file explorer (outside RStudio), find the file “GWASnetwork_discoverableGenesOnly.RData”. In Fig. 1, this was stored in sub-folder “objects”. Right-click the file, and press copy. In RStudio’s console, right-click and select paste. You may need to remove the prefix “file:///”. (B) In Mac finder, right click the file of interest (*control + click*). While the menu is open, press and hold the *alt/option* key. This reveals other options including **Copy “GWASnetwork_discoverableGenesOnly.RData” as Pathname.** Continuing to hold the *alt/option* key select the copy as pathname option. Then you can use *control + c* to paste the pathname. | |

- Step 2 – Apply thresholds to the network.

Once the network representation of the GWAS Catalog is loaded, enter desired thresholds for the Jaccard coefficient and the FDR adjusted p-value. The Jaccard coefficient is a measure of how much overlap there is between the genes of two nodes (each node represents all genes found to associate with a particular trait by a particular study) and can vary from 0 (no overlap) to 1 (complete overlap). A reasonable setting is a Jaccard coefficient greater than or equal to 0.5 and an FDR-adjusted p-value less than or equal to 0.05.

Links are present between nodes of the network if there is significant overlap in genes (see user manual for more detail).

What significance threshold do you want to filter the FDR adjusted p values by?

0.05

Small overlap (represented by small Jaccard coefficients) may be significant but irrelevant. What threshold do you want to filter the Jaccard coefficients (from 0 to 1) by?

0.5

- Step 3 – Explore search terms.

Next, search terms can be explored to decide on the most appropriate one. Another R window will pop up (Fig. 12). Here, we can type a term into the search bar and scan the returned results. Search terms can include spaces but capitalization is ignored. Any trait or study title that contains the search term will be returned. Scrolling to the bottom of the window, you can see how many search results are present. When done exploring, the window needs to be closed using the “Quit” button, not the “X” button windows are usually closed with. If the “X” button is used, the workflow will not detect that the window has been closed, and will not continue. In this case, RStudio needs to be closed and the workflow needs to be restarted.

The pop-up window offers a preview of results but it should be noted that the final search (next step) will only select exact matches whereas the preview window will also return individual words when more than one word is entered. For example, when previewing “cardiovascular disease”, results with cardiovascular and disease appearing separately are returned (though results with just disease are not returned). The final search term (next step) will only select exact matches to “cardiovascular disease”. This will result in 35 results rather than the 39 listed at the bottom of the preview window.

Do you want to explore search terms before selecting a final one (y or n)?

y

Another R window (called Shiny) will pop up. Use the search bar at the top right to try out search terms (note that capitalization is ignored). You can also use the search bars at the top of the columns to search a specific column. The number of results is mentioned at the bottom of the table. When you are finished, click the Quit button NOT the x button that closes the window.

Please press enter to continue...

Listening on http://127.0.0.1:5297

| 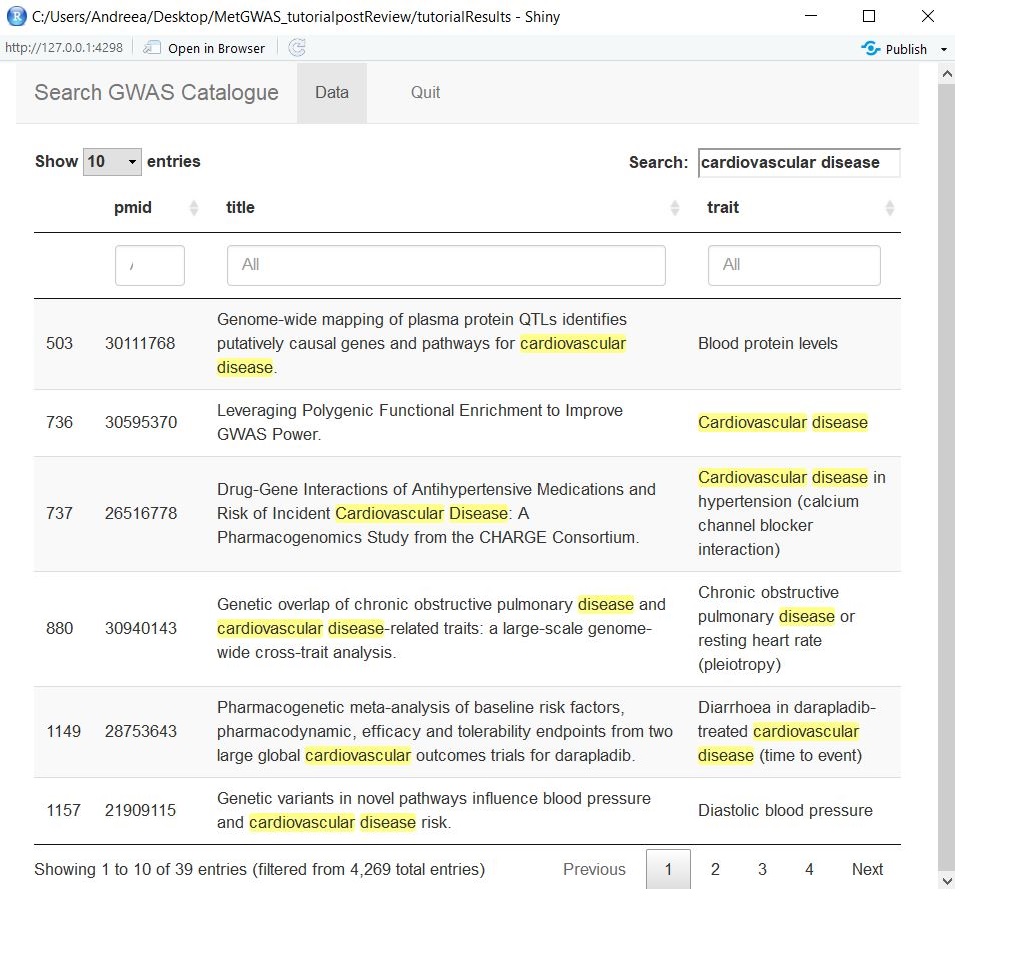  A |
| --- |
| 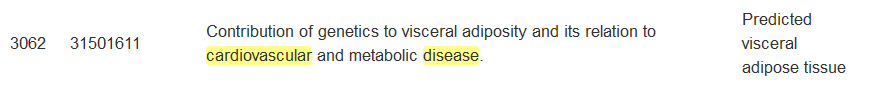  B |
| **Figure 12: An R window pops up to allow different search terms to be explored.** (A) From top to bottom, red circles mark the quit button, the main search bar and the number of entries returned by the search term inputted. For space purposes, the middle section of the results are not shown, indicated by the dotted red line. (B) An example returned in the preview window (on page 4) that would not be selected by the final search term because it is not an exact match to the search term. |

- Step 4 – Select a final search term.

The workflow will now prompt for the chosen search term. Use “cardiovascular disease” as a keyword search term. The workflow will select all nodes in the network containing the exact search term (ie nodes with study titles or trait names containing the search term) and all neighbouring nodes.

Please enter your chosen search term (note that capitalization is ignored):

cardiovascular disease

- Step 5 – Graph the GWAS Catalog network.

The workflow now gives the option (as long as there is more than 1 node in the network) of to graph and cluster the filtered GWAS network (Fig. 13). This can help visualize which traits are related to the searched trait.

Do you wish to graph/find clusters of the search terms' subnetwork (y or n)?

y

Clustering...

Graphing...

| 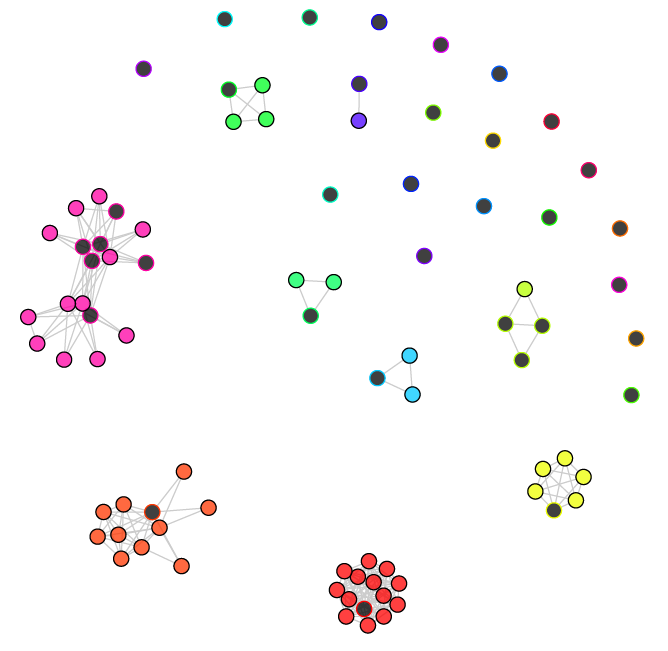 | 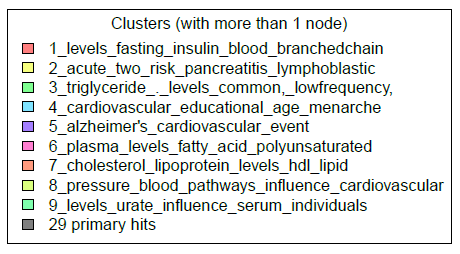 |
| --- | --- |
| **Figure 13: The cardiovascular disease network**. Colours denote which cluster a node belongs to. Nodes are coloured black if they contain the search term in their name (their edge colour then represents cluster membership). Clusters are named based on word frequency. Many studies on cardiovascular disease have no significant overlap with other nodes in the network (they are not connected) but those that do have overlap seem to connect to traits related to insulin and lipids. | |

Clusters are named after the five most frequent words in the names of nodes that belong to the cluster. If multiple words are tied for the fifth most frequent word, they are picked based on alphabetical order. This can be checked after saving results (see **Appendix F**).

- Step 6 – Save results.

Finally, the workflow will ask to save the graphs and the rest of the results. Saving results as the R object (or with both csv and R formats) is important if you intend to recreate the network graph and alter graphing parameters as the csv files cannot be used to create the graphic.

Do you wish to save the graph as a PNG (y or n)?

n

Do you wish to save the graph as a PDF (y or n)?

y

GWAS Genes module complete!

Do you wish to save the results of this module (y or n)?

y

If you prefer to save your data as csv files, it is recommended for this module that you also save the data in an RData file (ie option 3) should you need to re-graph the network.

How do you wish to save the results?

1 - as R objects in an RData file

2 - as csv files

3 - both

4 - neither

Enter a number:

3

Results were saved in D:\JohnDoe\MetGWAS_tutorial\tutorialResults

The csv files output by the workflow should match those in the “metGWAS results” sub-folder of Supplementary Materials 2 (Dataset 2 Tabassum). The csv file ending in “netGenes” contains all the genes present in the resulting network. This is the disease gene set and will be used in the following module. For more detailed descriptions of each file in the output, see **Appendix F**.

**OVER-REPRESENTATION ANALYSIS**

*Overview of Steps*

Module 4 - *Over-representation analysis for genetic predisposition*

- Step 1: Confirm the module is being run in the same source as modules 2 and 3.
- Step 2: For the hypergeometric test, provide a file path to the discoverable gene set.
- Step 3: Save results.

*Description of steps for over-representation analysis*

*Module 4 – Over-representation analysis for genetic predisposition*

- Step 1 – Confirm the module is being run in the same source as modules 2 and 3.

This module will compare the amount of overlap between metabolite-interacting genes found by module 2 and the trait/disease-related GWAS network (i.e., trait/disease-genes) found by module 3 from the full GWAS Catalog network. As such, it requires data from both module 2 and module 3. If the workflow is run immediately after these modules (ie in the same source), the workflow will automatically detect which data is to be used (for more details, see **Appendix C**). Enter “y” to proceed.

Do you wish to the “Disease Over-Representation” module (y or n)?

y

Running Disease Over-Representation module...

Are you running this module immediately after modules 2 (Interacting Proteins and Genes) and 3 (GWAS Genes)? In other words, was this workflow uninterrupted (y or n)?:

y

- Step 2: For the hypergeometric test, provide a file path to the discoverable gene set.

The workflow will now run a hypergeometric test for over-representation of the metabolite genes in the disease gene set and will return the upper tail p-value. Here, the workflow will ask for a path to the RData file beginning with “discoverableGenes” that came with this workflow (Fig. 14).

| 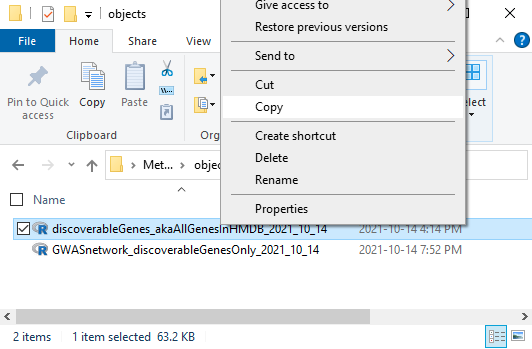  **A** | 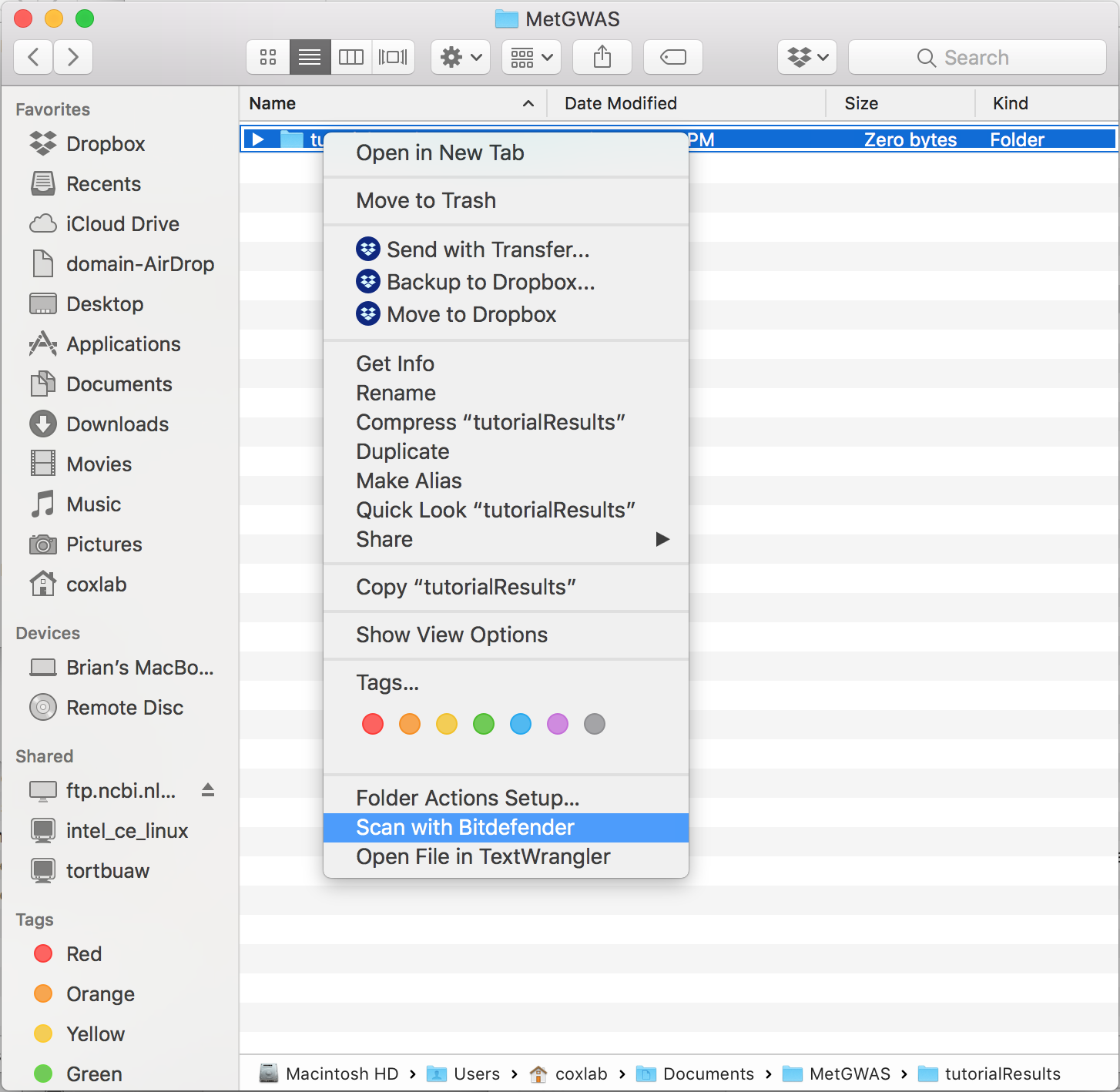  **B** |
| --- | --- |
| **Figure 14: File path to the discoverable gene set. (A)** In Windows file explorer (outside of RStudio), find the file “discoverableGenes_akaAllGenesInHMDB.RData”. In Fig. 1, this was stored in sub-folder “objects”. Right-click the file, and press copy. In RStudio’s console, right-click and select paste. You may need to remove the prefix “file:///”. (B) In Mac finder, right click the file of interest (*control + click*). While the menu is open, press and hold the *alt/option* key. This reveals other options including **Copy “discoverableGenes_akaAllGenesInHMDB.RData” as Pathname.** Continuing to hold the *alt/option* key select the copy as pathname option. Then you can use *control + c* to paste the pathname. | |

Please enter a file path to your discoverable gene set (an RData file):

D:/JohnDoe/MetGWAS_tutorial/objects/discoverableGenes_akaAllGenesInHMDB_2021_10_14.RData

Disease Over-Representation module complete!

Do you wish to save the results of this module (y or n)?

y

How do you wish to save the results?

1 - as R objects in an RData file

2 - as csv files

3 - both

4 - neither

Enter a number:

3

Results were saved in D:\JohnDoe\MetGWAS_tutorial\tutorialResults

- Step 3: Save results.

After asking to save results, the module and the workflow are both complete.

The csv files output by the workflow should match those beginning with “mod4” in the “metGWAS results” folder of Supplementary Materials 2. The csv file “mod4_overlap_genes” contains the genes that are found in both the metabolite gene set and the disease gene set. The csv file “mod4_overrep_pval” contains the number of discoverable genes followed by the p-value denoting if the overlap between the two gene sets is significant. For more detailed descriptions of each file in the output, see **Appendix F**.

REFERENCES

1. Tabassum, R. *et al.* Genetic architecture of human plasma lipidome and its link to cardiovascular disease. *Nat. Commun.* **10**, 1-14 (2019).
2. Xia, J., Psychogios, N., Young, N. & Wishart, D. S. MetaboAnalyst: A web server for metabolomic data analysis and interpretation. *Nucleic Acids Res.* **37**, 652–660 (2009).
3. Chong, J. *et al.* MetaboAnalyst 4.0: Towards more transparent and integrative metabolomics analysis. *Nucleic Acids Res.* **46**, W486–W494 (2018).
4. R Core Team, R: A language and environment for statistical computing. <http://www.R-project.org/> (2020).
5. RStudio Team. RStudio: Integrated Development for R. <http://www.rstudio.com/> (2016).
6. Morgan, M. BiocManager: Access the Bioconductor Project Package Repository. R package version 1.30.10. <https://CRAN.R-project.org/package=BiocManager> (2019).
7. Morgan, M. BiocVersion: Set the appropriate version of Bioconductor packages. R package version 3.10.1. <https://CRAN.R-project.org/package=BiocManager> (2019).
8. Harrison, J. RSeleium : R bindings for ‘Selenium WebDriver’. R package version 1.7.7. [https://CRAN.R-project.org/package=RSelenium](https://CRAN.R-project.org/package=RSelenium%20) (2020).
9. Ogata, H., *et al.* KEGG: Kyoto Encyclopedia of Genes and Genomes. *Nucleic Acids Res.* **27**, 29–34 (2009).
10. Wishart, D. S. *et al.* HMDB 4.0: The human metabolome database for 2018. *Nucleic Acids Res.* **46**, D608–D617 (2018).
11. Buniello, A. *et al.* The NHGRI-EBI GWAS Catalog of published genome-wide association studies, targeted arrays and summary statistics 2019. *Nucleic Acids Res.* **47**, D1005–D1012 (2019).

APPENDICES

*Appendix A: RStudio*

RStudio is an environment for using the computer language R. RStudio contains four sections, illustrated in Fig. 15 (note that layout can be edited). The two quadrants you will need to be familiar with are the window containing scripts and the window containing the console.

Scripts are files containing code that has been saved. Once in RStudio, to open a script, go to File > Open File and then select the R script desired. Pressing the source button will execute all the code in a script. This workflow for example, is run by sourcing the metGWAS.R_2021_12_01.R script. Output and prompts from the workflow will be displayed in the console. The console is also where you can enter input.

| 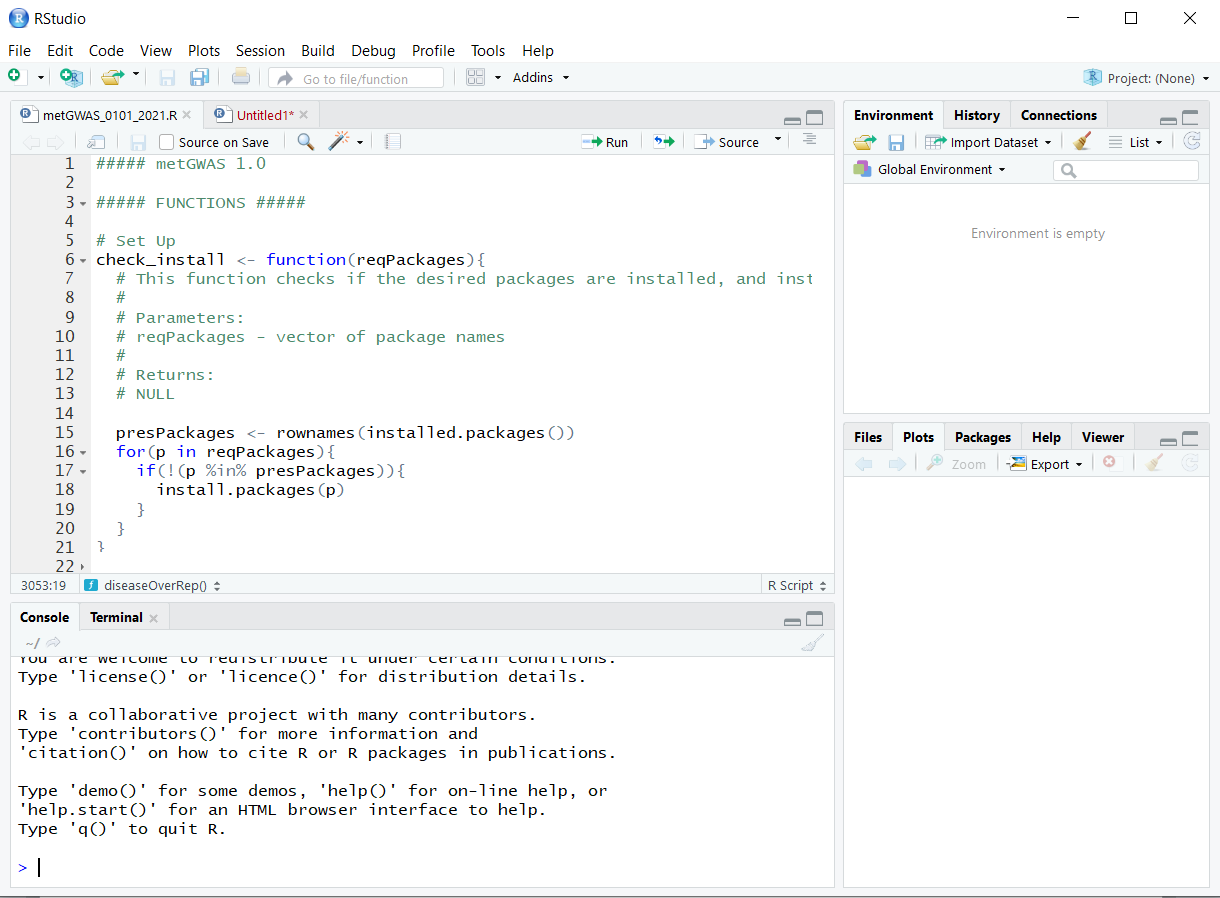 |
| --- |
| **Figure 15: RStudio layout.** The top left quadrant (red dashes) contains scripts. If multiple scripts are open at the same time, there will be multiple tabs in this window. Ensure you are on the appropriate tab, as the source button will only execute code in the selected tab. In this figure, pressing the source button (red circle) would run metGWAS_2021_12_01.R, it would not run the untitled script. To open a script, go to File (red square) > Open File and select the desired script (R file). The bottom left window (red dots) contains the console. Ensure you are on the console tab and not the terminal tab. |

*Appendix B: Using R objects as inputs for data.*

If inputting data as an R object, there are a few things to be aware of. Firstly, R objects need to be present in the environment before beginning the workflow. The exceptions to this are the objects in the three RData files deposited with the workflow (see Set-Up: Files Required), as these are reference datasets, not the data of interest. Secondly, the R object should not be named any of the following:

- res_pathOverRep
- res_interProtGenes
- res_GWAS
- res_diseaseOverRep

These are objects that will be created by modules 1 to 4 respectively. The modules will overwrite any objects with these names. Finally, data should be an object, not an element within an object. For example, data should not be a data frame within a list. Instead, it should be a data frame on its own.

*Appendix C: Running module “Y” immediately after module “X”.*

Each module stores results in a list object (see **Appendix B** for their names). Importantly, if a module is skipped, NULL objects with the same names are created. Whether a module is run or skipped, the resulting objects remain in the R environment even after the workflow has moved on to the next module.

The input for some modules is the output from previous modules. Module 2 uses “res_pathOverRep$responsibleMetabolites” from module 1 as input. Module 4 uses “res_interProtGenes$proteins$Primary Gene Names” from module 2 and “res_GWAS$rel_genes” from module 3 as input. In these instances, the workflow will ask if module “Y” is being run immediately after module “X” (ie the source button was only clicked once). If so, results from module “X” are already in the environment and with a particular name. The workflow therefore knows how to access the appropriate results. If module “Y” is not being run immediately after module “X”, the workflow will need to be given the appropriate input.

Results can be inputted with either a file path to a csv file or with the name of an R object. In the latter case, the R object needs to be present in the environment. This means it should have been loaded into the environment before the workflow was started. It should not have the same name as any of the four list objects created by the modules (see **Appendix B**). If it does, it will be overwritten when the workflow is run. The object also needs to be its own object, not an element in a list.

Even if module “Y” is run immediately after module “X”, you may not wish the workflow to use the results directly from module “X”. In some cases, you may make manual edits to the data before using it as input for the next module. These edits are easier to make in csv files, as edits to the R objects cannot be done without exiting the workflow. To use these edited files as inputs for module “Y”, the workflow should be told that module “Y” is not run immediately after module “X”.

*Appendix D: Updating the network representation of the GWAS Catalog.*

Note that a full discoverable GWAS Catalog network is provided in Supplementary Materials 1 (as “GWASnetwork_discoverableGenesOnly_2021_10_14.RData”). This was created off of the GWAS Catalog from December 2019. Over time, the Catalog grows with new information and it may be desirable to update the network representation. The workflow provides an option to do so when running module 3. This option also allows users to create network representations of the GWAS Catalog that are not limited to discoverable genes (genes present in HMDB). Such a network should only be used if the user’s sole interest is to search the GWAS Catalog for a trait or disease and the user does not compare the trait/disease to metabolites.

The amount of time it takes to update the network representation of the GWAS Catalog will depend firstly on whether the discoverable version or the network containing all genes is being updated. The former took about 3 hours while the latter took around 30 hours for the workflow to create when using the GWAS Catalog from December 2019. Over time, as the GWAS Catalog grows, this will increase the amount of time required. In both cases, the workflow needs to be provided with a file path to a tsv file containing the version of the GWAS Catalog you want to use. The latest GWAS Catalog can be downloaded at <https://www.ebi.ac.uk/gwas/docs/file-downloads>. Download “all associations v1.0”.

If updating the discoverable network, the workflow will also provide the option to update the discoverable gene set. This gene set is defined as the genes coding for all metabolite-interacting proteins in HMDB. Like the GWAS Catalog, this database can change over time, so the discoverable gene set should be updated periodically. The workflow will ask for a file path to a UniProt table and an HMDB table (Fig. 16). The UniProt table is used because it allows standardization of gene names to a primary name. The table can be downloaded from <https://www.uniprot.org/uniprot/?query=proteome:UP000005640>. Ensure the following columns are downloaded in the following order: Entry, Entry name, Status, Protein names, Gene names, Organism, Length, Gene names (primary), and Gene names (synonym). The HMDB table is used to filter for metabolite-interacting proteins and can be downloaded from <https://hmdb.ca/downloads>. If the discoverable gene set is not being updated, the workflow will ask for a file path to the existing discoverable gene set (the file “discoverableGenes_akaAllGenesInHMDB.RData”).

All objects created will be saved without prompts. The workflow will display the file names and directories where the objects are saved.

| 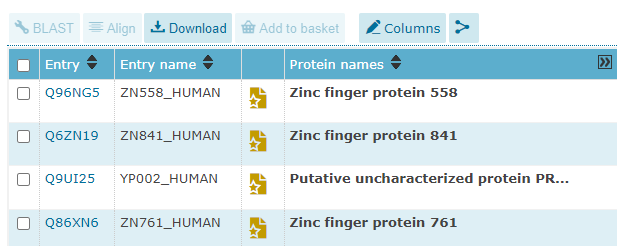  A | **Figure 16: Download tables for updating discoverable gene set.** (A) The UniProt table can be downloaded from the UniProt website by clicking the Download button (left red box). All columns displayed on the webpage are downloaded. These can be edited with the columns button (right red box). (B) The HMDB table can be downloaded from the HMDB website. Click download (red box) for the “All Proteins” data set (an XML file). |
| --- | --- |
| 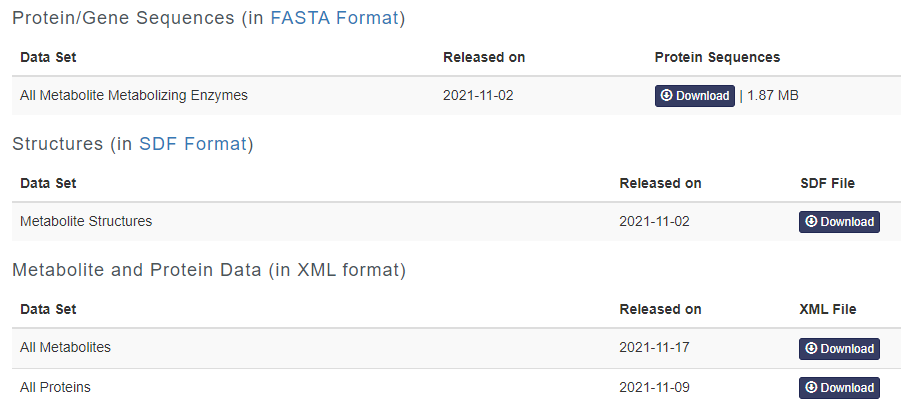  B |  |

*Appendix E: Advanced Usage.*

If you are new to R, it is recommended you start by sourcing the full script. If you are more experienced with R, the metGWAS 1.0 workflow provides a guide as to what steps should be carried out to analyze standalone metabolic data in conjunction with deposited GWAS data. You may wish to modify certain steps of the workflow.

All functions called in this workflow are placed at the top of the script (under the comment FUNCTIONS) and contain a description of the function. In the main part of the script (under the comment MAIN), each module is called to run by a single line of code:

res_pathOverRep <- pathOverRep() #module 1

res_interProtGenes <- interProtGenes() #module 2

res_GWAS <- gwasGenes() #module 3

res_diseaseOverRep <- diseaseOverRep() #module 4

When sourcing the script, each of the four modules will prompt to run. If you only want to run some modules and wish to avoid the prompts, you could run the single line of code in MAIN corresponding to the desired module. Before doing so, ensure all libraries have been loaded, all the functions have been created in the environment, and the chromeVersion and savePath objects have been created to ensure none of the modules throw an error. chromeVersion and savePath are character objects, and can be set in the following way:

chromeVersion <- setChrome() #part of MAIN

#or

chromeVersion <- “89.0.4389.23” #manual console input

savePath <- “D:/JohnDoe/Documents/Project” #manual console input

If unsure what to input as the chromeVersion, run the function setChrome and it will list possible chrome versions.

If manually inputting savePath, use forward slashes (in windows, copying and pasting file paths results in backslashes which will need to be changed). If sourcing the full script, when prompted for file paths, the slashes do not need to be forward, the readline function in the script will take care of them.

*Appendix F: Outputs.*

Results of each module can be saved as either an R object in RData files and/or csv files. In the former case, module results are placed in a single list object with different elements of the list representing different outputs. In the latter case, the results of a module are saved as several csv files (each file corresponding to an element of the R list object for that module). The following tables describe the outputs of each module (R object in the left column, csv files in the right column).

| **Table I: Data returned by module 1, either saved as mod1_res_pathOverRep.RData contents and/or equivalent csv files.** | |
| --- | --- |
| Element Name  (accessed by res_pathOverRep$element_name) | Csv File Name  (preceded by mod1_, ending with .csv) |
| Element Description | File Description |
| invesPaths | pathways_with_metabolites |
| Dataframe with rows of pathways and 8 columns:   1. “Total.Cmpd” is the total number of compounds in the pathway (including those not inputted by the user) 2. “Hits” is the number of inputted metabolites that belong to the pathway (may be updated from MetaboAnalyst’s original value) 3. “Raw.p” is the p value for over-representation of metabolites of interest in the pathway 4. “neg.log.p” is the negative log of the p value 5. “Holm.adj” is the Holm adjusted p value 6. “FDR” contains the FDR adjusted p value 7. “Impact” contains the impact of the pathway calculated by MetaboAnalyst 8. “hitNames” contains metabolites that were inputted by the user and belong to that pathway (metabolites are separated by “;”)   The pathway names are the rowname.  Note that all p values are calculated by MetaboAnalyst and are not updated to represent any extra hits that are found. | Rows of pathways and 9 columns:   1. the pathway name (no header for this column) 2. “Total.Cmpd” is the total number of compounds in the pathway (including those not inputted by the user) 3. “Hits” is the number of inputted metabolites that belong to the pathway (may be updated from MetaboAnalyst’s original value) 4. “Raw.p” is the p value for over-representation of metabolites of interest in the pathway 5. “neg.log.p” is the negative log of the p value 6. “Holm.adj” is the Holm adjusted p value 7. “FDR” contains the FDR adjusted p value 8. “Impact” contains the impact of the pathway calculated by MetaboAnalyst 9. “hitNames” contains metabolites that were inputted by the user and belong to that pathway (metabolites are separated by “;”)   Note that all p values are calculated by MetaboAnalyst and are not updated to represent any extra hits that are found. |
| responsibleMetabolites | metaboliteNames_HMDBcodes |
| Dataframe with rows of metabolites and 2 columns:   1. “metNames” is the inputted metabolite name (which could be an HMDB code) 2. “HMDBcode” is the corresponding HMDB code for the metabolite | Rows of metabolites with 2 columns:   1. “metNames” which is the inputted metabolite name (which could be an HMDB code) 2. “HMDBcode” which is the corresponding HMDB code for the metabolite |
| dups | metabolites_with_multipleHMDBcodes |
| Dataframe that is a subset of responsibleMetabolites and that contains only metabolites that appear more than once because they had multiple HMDB ids (may therefore be empty). | A subset of metaboliteNames_HMDBcodes file that contains only metabolites that appear more than once because they had multiple HMDB ids (may therefore be empty except of headers). |
| missingMet | cIdMissing |
| Character vector of inputted metabolites that did not have a C code. | A column of metabolites with the header “metabolites_with_no_c_number_annotation” |
| moreHits | pathways_with_extraHits |
| Character vector of pathway names that the workflow found more hits for than MetaboAnalyst did. Only generated when workflow uses MetaboAnalyst results in csv format as input. | A column (named “pathways_with_extra_hits_found”) of pathway names that had more metabolite hits found than what MetaboAnalyst found. Only generated when workflow uses MetaboAnalyst results in csv format as input. |

| **Table II: Data returned by module 2, either saved as mod2_res_interProtGenes.RData contents and/or equivalent csv files.** | |
| --- | --- |
| Element Name  (accessed by res_interProtGenes$element_name) | csv File Name  (preceded by mod2_, ending with .csv) |
| Element Description | File Description |
| priMetCodes | primaryMetaboliteCodes |
| Dataframe with rows of metabolites and 3 columns:   1. metabolites names given 2. “input” is the searched HMDB codes 3. “primary” is the primary HMDB codes | Rows of metabolites with 2 columns:   1. “input” which is the searched HMDB code 2. “primary” which is the primary HMDB code |
| proteins | proteinsGenes |
| Dataframe with rows of proteins and 5 columns:   1. “Name” is the enzyme name 2. “UniProt ID” is the UniProt id 3. “Primary Gene Name” is the gene name 4. “Type” is the type of protein (E.g. enzyme or unknown) 5. “Primary metCode” is all primary HMDB codes that had the enzyme listed under them(separated by “\t ”) | Rows of proteins with 5 columns:   1. “Name” which is the enzyme name 2. “UniProt ID” 3. “Primary Gene Name” which is the gene name 4. “Type” which refers to the type of protein, for example, Enzyme or Unknown 5. “Primary metCode” which is all the HMDB codes searched that had the enzyme listed (all in a single cell) |
| noHMDBPage | checkMetaboliteHMDB* |
| Character vector of HMDB codes that were not found on HMDB site. | A single column of HMDB codes that did not have a page on HMDB (column name is “x”). |
| noProteins | metabolitesWithoutProteins* |
| Character vector of HMDB codes that had no associated proteins. | A single column of HMDB codes which had no associated proteins (column name is “x”). |
| *Saved only if corresponding R object is not empty. | |

| **Table III: Data returned by module 3, either saved as mod3_res_GWAS.RData contents and/or equivalent csv files.** | |
| --- | --- |
| Element Name  (accessed by res_GWAS$element_name) | Csv File Name  (preceded by mod3_searchTerm_from_# where search term is what the GWAS catalogue was filtered for and # is the number of genes in the full GWAS catalogue before filtering for the search term) |
| Element Description | File Description |
| n_all_genes | no equivalent file (but part of other file names) |
| Integer representing total number of genes in the GWAS network (before filtering for search term). |  |
| searchTerm | no equivalent file (but part of other file names) |
| The search term that was entered to filter the GWAS network. |  |
| rel_nodeNames | netNodeNames |
| Dataframe with rows of node names and 2 columns:   1. “nodes” which gives the name of the node (in the format pubmedID_studyTitle_traitStudied) 2. “hitType” denotes how the node passed the search filter (1 if node name contained search term, 2 if node was a neighbor to one containing the search term) | Rows of node names and 2 columns:   1. “nodes” which gives the name of the node (in the format pubmedID_studyTitle_traitStudied) 2. “hitType” denotes how the node passed the search filter (1 if node name contained search term, 2 if node was a neighbor to one containing the search term) |
| rel_genes | netGenes |
| Character vector of gene names representing all the genes in the GWAS network after filtering for the search term (note that each gene appears only once). | A single column of gene names representing all the genes in the GWAS network after filtering for the search term (column name is “x”). |
| relNet | no equivalent file |
| igraph object of the GWAS network after filtering for the search term. |  |
| c_relNet | no equivalent file |
| igraph object from clustering relNet. |  |
| c_summary | clusterSummary |
| Dataframe with rows of clusters and 6 columns:   1. “cluster” is the cluster number 2. “name” is the name of the cluster based on searching for the most common words in the node names within the cluster 3. “total_nodes” is the number of nodes in the cluster 4. “primary_hits” is how many of the nodes in the cluster had the search term in their name 5. “perc_of_c” is the percentage of the nodes in the cluster that are a primary hit 6. “perc_of_primary” is the percentage of primary hit nodes from the full network that are found in the cluster | Rows of clusters with 6 columns:   1. “cluster” is the cluster number 2. “name” is the name of the cluster based on searching for the most common words in the node names within the cluster 3. “total_nodes” is the number of nodes in the cluster 4. “primary_hits” is how many of the nodes in the cluster had the search term in their name 5. “perc_of_c” is the percentage of the nodes in the cluster that are a primary hit 6. “perc_of_primary” is the percentage of primary hit nodes from the full network that are found in the cluster |
| c_patterns | clusterNamePatterns |
| List where each element represents a cluster and the word patterns found in that cluster using a data frame with 2 columns (“word” and “freq” for frequency with which the word was found). | Each sheet represents a cluster with 2 columns, “word” and “freq” representing the word patterns found for that cluster. |
| c_nodes | clusterNodes |
| Dataframe with each column representing a cluster and filled with the names of nodes belonging to that cluster (note that the columns are named after the cluster names only if the cluster has more than one node). | Column representing a cluster and filled with the names of nodes belonging to that cluster (note that the columns are named after the cluster names only if the cluster has more than one node, otherwise they are named NA). |
| c_genes | clusterGenes |
| Dataframe with each column representing a cluster and filled with the gene names that belong to that cluster (genes only appear once even if they belong to multiple nodes). | Each column represents a cluster and is filled with the gene names that belong to that cluster (genes only appear once even if they belong to multiple nodes). |
| subnetwork_graph | subnetwork (pdf or png) |
| recordedPlot object of the network graph filtered for the search term. Colours denote cluster membership. Note that colours may be repeated if there are many clusters, though the colours are more likely to be unique between the largest clusters. Furthermore, the legend only displays clusters with more than one node. Nodes that were primary hits are black. | Colours denote cluster membership. Note that colours may be repeated if there are many clusters, though the colours are more likely to be unique between the largest clusters. Furthermore, the legend, which is on the second page of the pdf, only displays clusters with more than one node. Nodes that were primary hits are black. |

| **Table IV: Data returned from module 4, saved as either mod4_res_diseaseOverRep.RData contents and/or equivalent csv files.** | |
| --- | --- |
| Element Name  (accessed by res_diseaseOverRep$element_name) | Csv File Name  (preceded by mod4_, ending with .csv) |
| Element Description | File Description |
| overlap_genes | overlap_genes |
| Character vector of gene names that were both in the metabolite and disease gene sets produced by modules 2 and 3 respectively. | One column (“genes_metResults_and_diseaseResults”) containing gene names that were in both the metabolite and disease gene sets. |
| overrep_stat | overrep_pval |
| p value (upper tail) of the hypergeometric test that determines if overlap between the metabolite gene set and disease gene set is significant. | the number of background genes followed by the p value (upper tail) of the hypergeometric test that determines if overlap between the metabolite gene set and disease gene set is significant. |
| n_background | no equivalent file (part of overrep_pval file) |
| The number of discoverable genes. |  |
