# Supplementary material for: metGWAS 1.0: an R workflow for network-driven over-representation analysis between independent metabolomic and meta-genome-wide association studies
Source: Bioinformatics. 2023 Aug 23;39(9):btad523. doi: 10.1093/bioinformatics/btad523 (PMC10491949; doi:10.1093/bioinformatics/btad523)
Supplement: btad523_Supplementary_Data [file btad523_supplementary_data.zip › Supplementary Material 2/Case Study-1_Tabassum et al/Workflow restults/mod3_ cardiovascular disease_from_4122 _subnetwork.pdf]

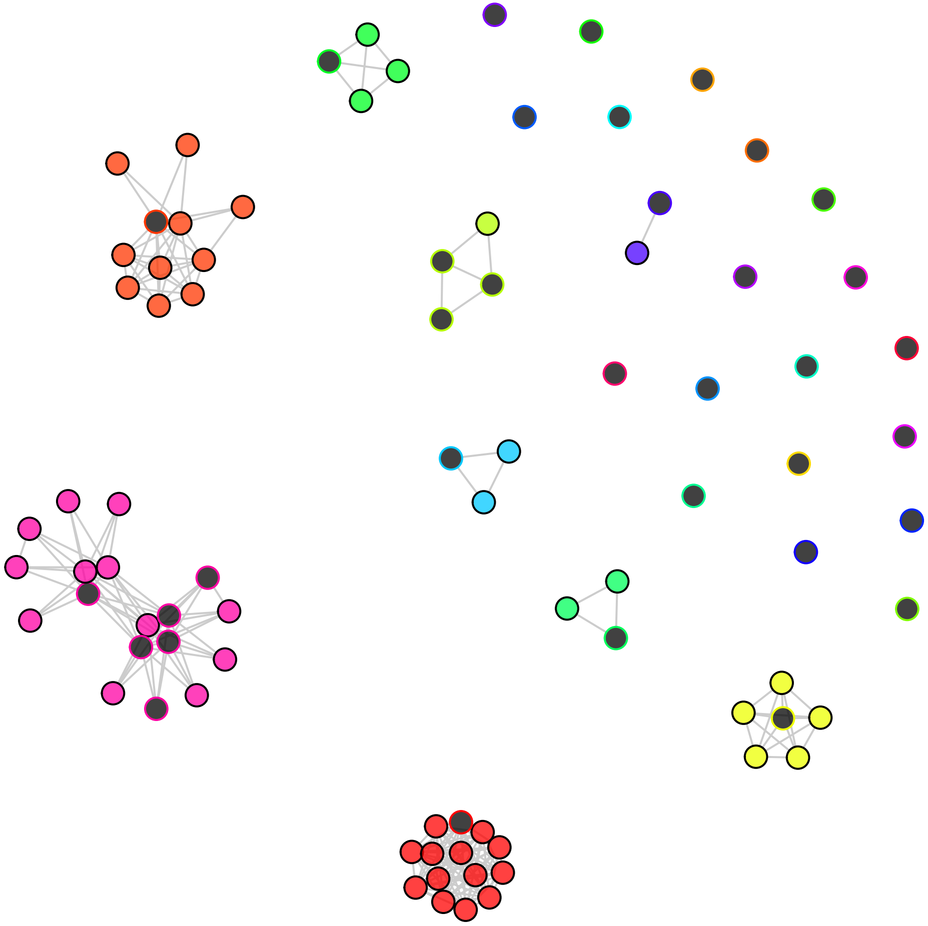

Clusters (with more than 1 node)

- 1_levels_fasting_insulin_blood_branchedchain
- 2_acute_two_risk_pancreatitis_lymphoblastic
- 3_triglyceride_._levels_common,_lowfrequency,
- 4_cardiovascular_educational_age_menarche
- 5_alzheimer's_cardiovascular_event
- 6_plasma_levels_fatty_acid_polyunsaturated
- 7_cholesterol_lipoprotein_levels_hdl_lipid
- 8_pressure_blood_pathways_influence_cardiovascular
- 9_levels_urate_influence_serum_individuals
- 29 primary hits
