# Supplementary material for: metGWAS 1.0: an R workflow for network-driven over-representation analysis between independent metabolomic and meta-genome-wide association studies
Source: Bioinformatics. 2023 Aug 23;39(9):btad523. doi: 10.1093/bioinformatics/btad523 (PMC10491949; doi:10.1093/bioinformatics/btad523)
Supplement: btad523_Supplementary_Data [file btad523_supplementary_data.zip › Supplementary Material 2/Case Study-2_Suhre et al/workflow results/kidney/mod3_ kidney_from_4122 _subnetwork.pdf]

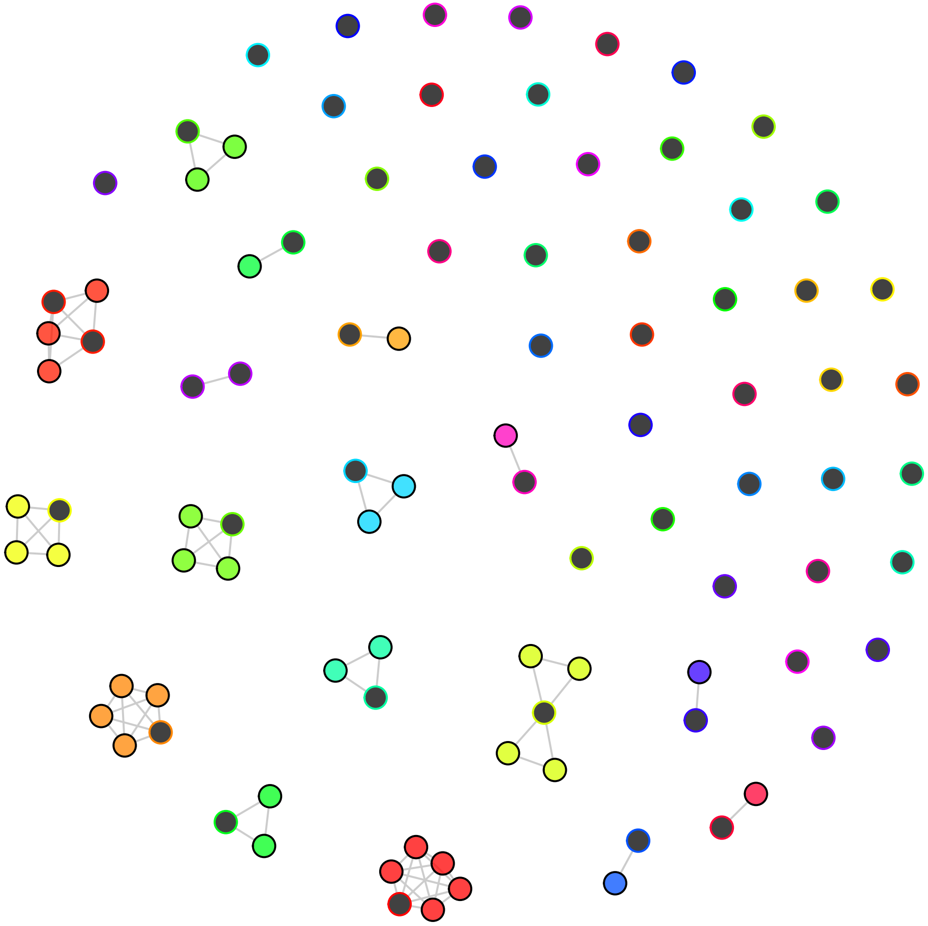

Clusters (with more than 1 node)

1_acute_two_risk_pancreatitis_lymphoblastic
2_alcohol_aldh2_associated_dependence_major
3_rapid_naming_alternating_stimulus_multivariate
4_childhood_acute_lymphoblastic_leukemia_neutropenia/leucopenia
5_alzheimer's_type_1
6_type_2_diabetes_dna_methylation
7_skin_acute_kidney_injury_coronary
8_renal_type_1_stage
9_bipolar
10_type_2_diabetic_kidney_subjects
11_kidney_interaction_dust_mite_allergen
12_brain
13_uric_acid_serum_levels_gout
14_multiple_functionrelated_traits_renal_gout
15_blood_pressure_smoking_diastolic_genetics
16_hyperuricemia_identification_cdc42bpg_susceptibility_japanese
17_metabolite
58 primary hits
