# Supplementary figures and images for: metGWAS 1.0: an R workflow for network-driven over-representation analysis between independent metabolomic and meta-genome-wide association studies
Source: Bioinformatics. 2023 Aug 23;39(9):btad523. doi: 10.1093/bioinformatics/btad523 (PMC10491949; doi:10.1093/bioinformatics/btad523)

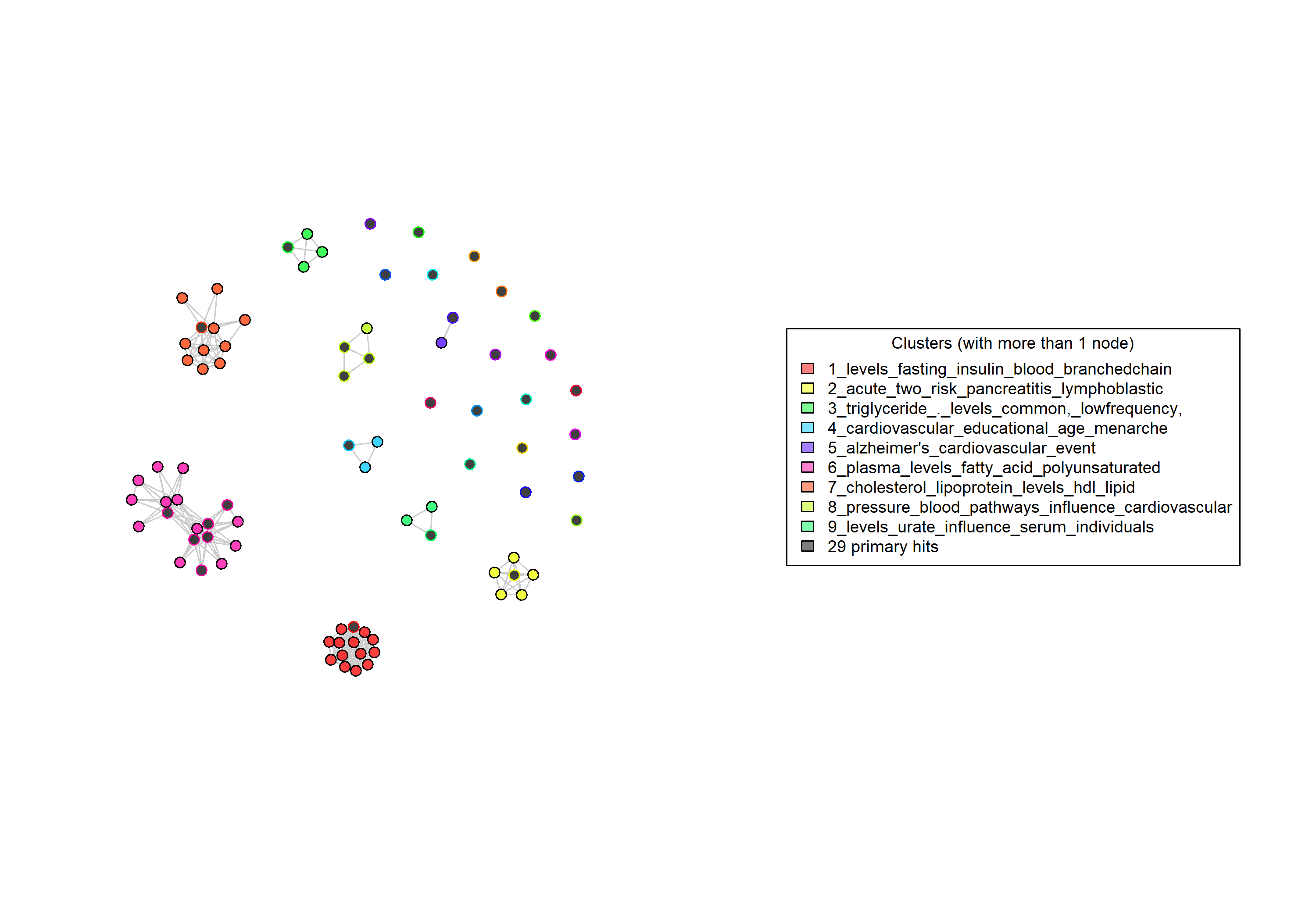

Supplement: btad523_Supplementary_Data [file btad523_supplementary_data.zip › Supplementary Material 2/Case Study-1_Tabassum et al/Workflow restults/mod3_ cardiovascular disease_from_4122 _subnetwork.png]

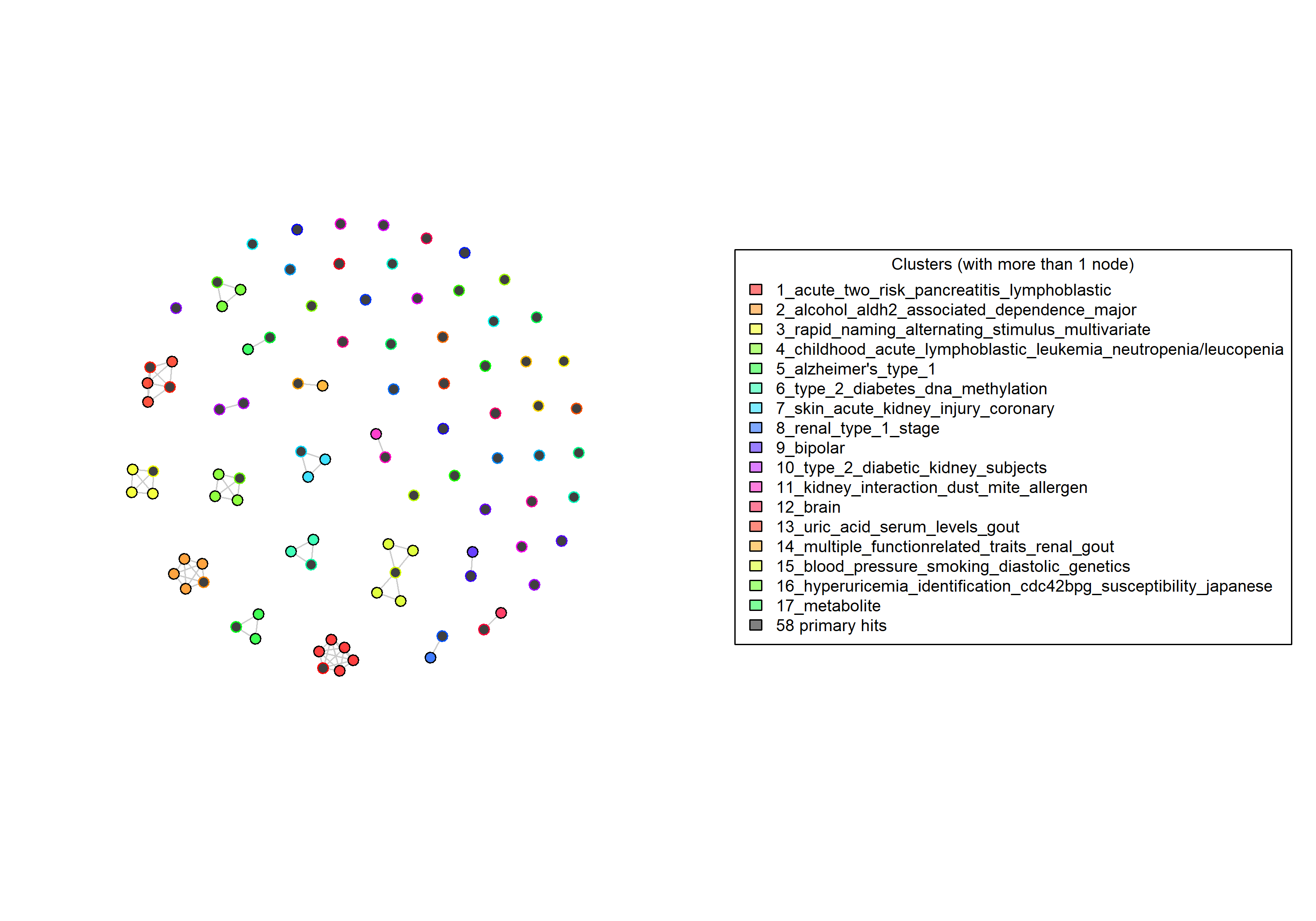

Supplement: btad523_Supplementary_Data [file btad523_supplementary_data.zip › Supplementary Material 2/Case Study-2_Suhre et al/workflow results/kidney/mod3_ kidney_from_4122 _subnetwork.png]
